# *Glycyrrhiza uralensis* extract supplementation mitigated the negative effects of prolonged low-dose exposure to Deoxynivalenol and Zearalenone on growth performance and intestinal health of broiler chickens

**DOI:** 10.3389/fvets.2025.1570265

**Published:** 2025-04-11

**Authors:** Yan Chen, Guohua Zhang, Jiawei Li, Ximei Li, Susu Jiang, Yingpai Zha Xi, Yanli Guo, Jianxiong Lu

**Affiliations:** ^1^College of Animal Science and Technology, Gansu Agricultural University, Lanzhou, China; ^2^School of Life Sciences and Engineering, Northwest Minzu University, Lanzhou, China

**Keywords:** *Glycyrrhiza uralensis* extract, broiler chickens, intestine health, growth performance, DON, ZEN

## Abstract

Deoxynivalenol (DON) and Zearalenone (ZEN), common symbiotic mycotoxins found in mold-contaminated cereal feed, adversely affect broiler’ health. *Glycyrrhiza uralensis* has various pharmacological effects including antibacterial, antioxidant and immunomodulatory. This study aimed to investigate the effects of the long-term intake of low doses of DON and ZEN on growth performance and intestinal health of broilers, as well as the potential protective effect of supplementary *Glycyrrhiza uralensis* extract (GUE) in an 84-day feeding experiment. A total of 315 one-day-old male *Liangfeng* broilers were randomly assigned to three treatments: basal diet (CON), MOL diet (where 5% of corn in the basal diet was replaced with an equal amount of naturally moldy corn) containing DON and ZEN at 1.25 and 1.29 mg/kg, and MGUE diet supplemented with 0.1% GUE in the MOL diet. The MOL diet reduced the body weight (BW) of broilers at 56 and 84 day, body weight gain (BWG) and feed intake (FI) aged 1-56 and 1-84 days, and the feed conversion ratio (FCR) aged 1-84 days, as well as villus height (VH) and the villus/crypt (V/C) ratio, SOD and GSH-Px activities, and the expression of *claudin-1*, *occludin* and *ZO-1*, while increasing MDA level, the expression of *TNF-α*, *IL-1β* and *IFN-γ* in the jejunum of broilers. Additionally, MOL diet decreased the *Firmicutes* to *Bacteroidetes* (F/B) ratio and abundances of *Lactobacillus* (*L.gallinarum* and *L.crispatus*), and *B.vulgatus*, while increasing *Bacteroides* (*B.fragilis* and *B.dore*), *Helicobacter* (*H.pullorum*), and *Escherichia* (*E.coli*) in the ceca. In contrast, MGUE diet improved growth performance and returned it to a level comparable to that of the CON diet, increased VH and V/C ratio, SOD and GSH-Px activity, *claudin-1*, *occludin* and *ZO-1* expression, while reducing MDA level, the expression of *TNF-α*, *IL-1β* and *IFN-γ* in the jejunum. Moreover, MGUE diet had a greater F/B ratio and abundance of *Lactobacillus* (*L.gallinarum* and *L.crispatus*) and *B.vulgatus*, while reducing *Bacteroides* (*B.fragilis* and *B.dorei*), *Helicobacter* (*H.pullorum*) and *Escherichia* (*E.coli*) in cecum. In conclusion, the long-term consumption of a low-dose DON-ZEN contaminated diet decreases growth performance and disrupts intestinal health and microbiota balance in broilers; however, dietary supplementation with GUE effectively mitigates the damage caused by DON-ZEN contamination.

## 1 Introduction

Mycotoxins are prevalent in nature, and feed in various stages of production, including cultivation, transportation, processing and storage, can be contaminated by mycotoxins. Approximately 25% of the world’s food crops are tainted with mycotoxins (1). The numerous types of low molecular weight mycotoxins, which are secondary metabolites produced by molds, are frequently present in feed ingredients, posing a threat to the health and production performance of farm animals (2), and the presence of mycotoxin residue in animal products was potentially hazardous to the health of humans (3). Mycotoxin contamination in poultry feed is a widespread issue. It is estimated that over 60% of feed ingredients worldwide contain detectable levels of mycotoxins, with Aflatoxins, Fumonisins, Deoxynivalenol (DON), and Zearalenone (ZEN) being the most common in maize-based poultry diets (4). A survey conducted in sub-Saharan Africa has identified multiple mycotoxin contaminations in poultry feeds, which have a detrimental impact on the health and productivity of the poultry industry, leading to significant economic losses (5). In developing countries, inadequate storage infrastructure and humid climates worsen mycotoxin contamination, resulting in annual economic losses of 1.5–3.0 billion US dollars in the poultry sector alone (6). DON and ZEN, the most common symbiotic mycotoxins (7), are recognized as the most widespread *Fusarium* contaminants in animal diets and feed ingredients (8). Both DON and ZEN can contaminate a majority of cereal crops during pre-harvest or post-harvest conditions (3). Exposure to DON and ZEN resulted in liver dysfunction and hepatocyte apoptosis (9), stimulated the synthesis of pro-inflammatory cytokines, disrupted normal immune responses (10), and led to abnormal morphology along with decreased progesterone and estrogen levels in ovarian granulosa cells (11). The consumption of feed contaminated with DON caused cytotoxic effects on enterocytes, leading to injury of intestinal barrier function and an increase in the permeability of the intestinal wall in broiler chickens (12). The barrier dysfunction not only facilitated the translocation of systemic toxins but also triggered local and systemic inflammation in animals (13), exacerbating production losses (14). Exposure to ZEN affected the digestive system of animals, resulting in a disruption of epithelial cell integrity and function (15).

The methods for decontaminating mycotoxins in feed products are diverse, including a range of physical and chemical techniques, along with biodegradation and biosorption (16). Dietary supplementation of catalase alleviated DON-induced oxidative stress and intestinal damage in broilers (17). *Silybum marianum* seed, *Thymus vulgaris*, and *Rosmarinus officinalis* powder reduced the risks of aflatoxin B1 (AFB_1_) in young broiler chicks (18). *Baicalin* protected against ZEN-induced liver and kidney injury in chicks by reducing oxidative stress, and modulating the caspase signaling pathway (19). However, low doses of mycotoxins contamination are widespread. A survey report on mycotoxin contamination indicated that the detection rates of aflatoxin ZEN and DON exceeded 90% in compound feed and raw material samples from various provinces and regions of China during 2019 and 2020 (20). Furthermore, the duration of exposure to mycotoxins is an important variable that affects their toxic effects (21). The gut serves as the initial and most sensitive biological target during chronic low-dose exposure to DON and other type B *Trichothecenes* (22). Dysfunction of the intestinal barrier can challenge the immune system and disrupt the host-microbial balance, initiating gastrointestinal and extra-intestinal disorders. Therefore, it is important to consider how to prevent potential harm to animal health, especially the intestinal barrier, from chronic exposure to low levels of mycotoxins.

*Glycyrrhiza uralensis* Fisch (licorice), a traditional medicinal and edible plant (23), has a long history of application in diets, beverages, and medicinal remedies (24). *Glycyrrhiza uralensis* extract (GUE) is derived from its dried stems and roots and contains a variety of bioactive compounds, including glycyrrhizin, glycyrrhizinic acid, glabridin, glabrene, and glabrol. Numerous studies have demonstrated that GUE possesses various pharmacological effects, such as antitumor (25), antibacterial (26), antioxidant (27) and immunomodulatory (25). Dietary supplementation of GUE upregulated the expression of tight junction protein occludin and Junctional Adhesion Molecule 2 (JAM-2), positively affecting the maintenance of intestinal integrity in broilers (28) and pigs (29). GUE has potential beneficial effects on gut barrier function (30), and dietary supplementation with *Glycyrrhiza uralensis* polysaccharides increased the diversity and altered the composition of cecal microbiota in broilers (31). Our previous study also demonstrated that GUE had a significant positive effect on the growth performance and health of broilers (32). In the present study, we investigated the impact of a long-term diet incorporating naturally moldy corn with low doses of DON and ZEN on the growth performance and intestinal health of *Liangfeng* broilers (a medium-growing broiler in China) and evaluated the potential protective effect of dietary supplementation with GUE against the damage caused by DON and ZEN. This study aims to provide a measure for preventing and mitigating the impact of dietary mycotoxin contamination on the growth and health of broiler chickens.

## 2 Materials and methods

The care and management of broilers and experimental procedures applied in this study were approved by the Institutional Animal Care and Use Committee of the Gansu Agricultural University (GsAu-Eth-AST-20210430).

GUE (purity>98%; glycyrrhizin≥10%; prepared from the root of *Glycyrrhiza uralensis*; Gansu YaLan Pharmaceutical Co., Ltd., China). The naturally moldy corn was supplied by farmers in Yuzhong County, Gansu, China. Before preparing the experimental diets, the mycotoxin content in the moldy corn was detected by Pony Testing International Group (Qingdao, China). The concentrations of DON, ZEN, and Fumonisin B (FB) were 25.70 ± 0.14, 25.50 ± 0.71, and 1.47 ± 0.01 mg/kg, respectively.

### 2.1 Experimental design and animal management

A total of 315 one-day-old healthy male *Liangfeng* broiler chickens (a medium-growing chicken) were randomly divided into three groups, with seven replicates per group and 15 broilers per replicate. The three dietary treatments were as follows: (1) basal diet (CON group), (2) the 5% corn in the basal diet was replaced by an equal amount of the moldy corn based on its DON and ZEN contents (33) (MOL group), (3) the MOL diet supplemented with 0.1% GUE (32, 34) (MGUE group). The basal diet (Table 1) (32) was formulated according to “Feeding Standard of Chicken in China (NY/T 33-2004)” issued by the Ministry of Agricultural of People’s Republic of China. The contents of DON, ZEN, and FB in both the MOL and MGUE diet were 1.25, 1.29, and 0.07 mg/kg, respectively (maximum allowance in broiler diets is ≤3.0, 0.5, and 20 mg/kg, respectively; GB13078-2017, China).

**Table 1:** Composition and nutrient levels of basal diets (air-dry basis).

Items	Starter 1 to 28 days of age	Grower-finisher 29 to 84 days of age
Ingredient (%)		
Corn	55.00	57.48
Soybean oil	2.90	4.20
Soybean meal	29.00	24.00
Cottonseed meal	1.50	1.70
Rapeseed meal	2.18	2.80
Corn gluten meal	6.90	6.60
CaHPO_4_	1.80	2.50
NaCl	0.30	0.30
L-Lys HCL	0.15	0.11
DL-Met	0.00	0.08
Cys	0.07	0.03
Premix^a^	0.20	0.20
Total	**100.00**	**100.00**
Nutrient levels^b^		
ME/(MJ/kg)	12.01	12.36
CP (%)	21.15	19.31
Ca (%)	0.85	0.91
TP (%)	0.60	0.62
Lys (%)	1.15	1.00
Met (%)	0.70	0.40

a Provides per kg of diet: Fe 80 mg; Cu 8 mg; Mn 80 mg; Zn 60 mg; I 0.35 mg; Se 0.15 mg; VA 8000 IU; VD3 1,000 IU; VE 20 IU; VK3 0.5 mg; VB1 2.0 mg; VB2 8.00 mg; VB6 3.50 mg; VB12 0.01 mg; Niacin 35.00 mg, D-pantothenate 10.00 mg; Folic acid 0.55 mg; Biotin 0.18 mg.

b Nutrient levels were all measured values.

The experiment was performed in Gansu Agricultural Vocational Farm Co. in Gansu, China. The temperature regimen was 34°C from days 1 to 14 and then gradually decreased by 2°C weekly to a final temperature of 26°C. The humidity was kept between 40 and 60% throughout the entire experiment, and the lighting regime was 24 h for the first week, then reduced to 16 h until the end of the experiment. All broilers had ad libitum access to feed and water. The experiment lasted for 84 days. The broilers were vaccinated with Newcastle disease vaccine and the infectious bursal polyvalent vaccine on d 7 and 14 of the experiment, respectively.

### 2.2 Growth performance

The body weight (BW) of all broilers was measured after fasting for 12 h every 2 weeks, and the feed intake on a replicate basis was recorded daily. The BW gain (BWG), feed intake (FI) and feed conversion rate (FCR, feed/gain) were calculated. Mortality was recorded as it occurred.

### 2.3 Sample collection

On the last day of the starter stage (days 1 to 28), the grower stage (days 29 to 56), and the finisher stage (days 57 to 84), that was on day 28, 56 and 84, two broilers were randomly selected from each replicate and were slaughtered by severing the jugular vein after a 12 h fasted feeding. The abdomen was disinfected with 75% ethanol, then immediately dissected. The entire intestine was carefully removed from the abdominal cavity, and the jejunum and cecum were separated with a sterile scalpel. Approximately 5 cm segments of jejunum from the posterior side of Meckel’s diverticulum were excised and divided into two parts after being rinsed with ice-cold phosphate-buffered saline (PBS; pH 7.4). One segment was immediately fixed in a 4% paraformaldehyde solution for paraffin sectioning, while the other was longitudinally cut, and the mucosa was gently scraped into an RNase-free tube using a sterilized glass slide, then snap-frozen in liquid nitrogen and stored at −80°C for the determination of antioxidant indices and RNA extraction. The cecal contents were carefully collected, and immediately homogenized, transferred to cryogenic vials, snap-frozen in liquid nitrogen and stored at −80°C until they were processed for microbial DNA analysis.

### 2.4 Intestinal antioxidant analysis

The enzyme activities of superoxide dismutase (SOD; Detection limit = 0.5 U/g) and glutathione peroxidase (GSH-Px; Detection limit = 0.2 nmol/min/g), and the malondialdehyde (MDA; Detection limit = 0.1 nmol/g) content in the jejunum mucosa were measured by a colorimetric method using kits according to the manufacturer’s protocol (Cell Biolabs Inc., San Diego, CA, United States).

### 2.5 Histopathological analysis

Jejunum segments fixed in paraformaldehyde for 24 h were dehydrated in ethyl alcohol for 24 h, cleared with xylene, and embedded in paraffin. The paraffin blocks were sectioned into 5-μm-thick tissue slices and stained with hematoxylin–eosin (H&E). The histological changes were examined using a light microscope (Leica, Germany), and ImageJ analysis software (National Institutes of Health, DC, United States) was employed to measure the villi height and crypt depth. Subsequently, the ratio of villus height to crypt depth (VH/CD) was calculated.

### 2.6 Real-time PCR analysis

Total RNA was extracted using RNAiso Plus (TaKaRa CAS. No. 9190). RNA integrity and purity were assessed using a NanoDrop One spectrophotometer. cDNA was synthesized with a PrimeScript TMRT Master Mix (TaKaRa Biotechnology, Dalian, China). Amplification was carried out in a total volume of 10 μL containing 5 μL of SYBR Green PCR Master Mix (SparkJade, Shandong, China), 1 μL of cDNA, 0.4 μL of each forward and reverse primer, and 3.2 μL RNase Free ddH_2_O. qRT-PCR was performed on a LineGene9660 real-time PCR system (BIOER, Hangzhou, China) with the following reaction condition: 95°C for 30 s, followed by 40 cycles of 95°C for 5 s, 60°C for 30 s, and 72°C for 30 s. The elative mRNA expression of target genes was calculated using 2^−ΔΔCt^ method and *β-actin* as an internal control. The information of primers used in this study was listed in Table 2.

**Table 2:** Primers for RT-qPCR analysis.

Gene	Accession number	Forward primers (5′-3′)	Reverse primers (5′-3′)
Occludin	XM_025144247.2	ACGGCAGCACCTACCTCAA	GGGCGAAGAAGCAGATGAG
ZO-1	XM_040706827.2	CTTCAGGTGTTTCTCTTCCTCCTC	CTGTGGTTTCATGGCTGGATC
Claudin-1	NM_001013611.2	CATACTCCTGGGTCTGGTTGGT	GACAGCCATCCGCATCTTCT
TNF-α	NM_204267.2	GAGCGTTGACTTGGCTGTC	AAGCAACAACCAGCTATGCAC
IL-1β	NM_204524.2	ACTGGGCATCAAGGGCTA	GGTAGAAGATGAAGCGGGTC
INF-γ	NM_205149.2	AGCTGACGGTGGACCTATTATT	GGCTTTGCGCTGGATTC
Actin	NM_205518.2	TCCACCGCAAATGCTTCTAA	TCCACCGCAAATGCTTCTAA

### 2.7 16S rDNA sequencing of cecal microbiota

The total microbial DNA of cecal content samples were extracted with the TGuide S96 kit (DP812; TIANGEN BIOTECH CO., LTD.) according to manufacturer’s instructions. The purity and quality of DNA were evaluated by the agarose gel electrophoresis. The V1–V9 region of the bacterial 16S rRNA gene was amplified using the forward primer 27 F (5’-AGRGTTTGATYNTGGCTCAG-3′) and the reverse primer 1,492 R (5’-TASGGHTACCTTGTTASGACTT-3′). Subsequently, the SMRTbell libraries were constructed by SMRTbell Express Template Prep Kit 2.0 (Pacific Biosciences, CA, United States), and sequenced on the PacBio Sequel II platform (Beijing Biomarker Technologies Co., Ltd., Beijing, China). The raw sequencing reads were processed to obtain Circular Consensus Sequencing (CCS) sequence using the SMRT Link software v8.0. The CCS sequence was divided into each sample according to sample barcodes, and chimera sequences were removed to obtain the clean reads using the UCHIME algorithm v8.1. Operational taxonomic units (OTUs) were clustered at a 97% similarity threshold using USEARCH v10.0. The SILVA database was used to taxonomically annotate OTUs with a confidence threshold of 70%.

The alpha diversity indices, Shannon and ACE, were calculated and presented for each treatment to illustrate the richness and uniformity of microbial communities using QIIME2 software. Beta diversity was assessed using Partial Least Squares Discriminant Analysis (PLS-DA) and Principal Coordinate Analysis (PCoA) based on the weighted-Unifrac distance matrix with QIIME software, which was employed to demonstrate the degree of similarity of microbial communities among groups. Analysis of Variance was used to examine differences in the relative abundance of microbial communities at different taxonomic levels (phylum, genus and species). Furthermore, Linear Discriminant Analysis (LDA) combined with effect size measurements (LEfSe) was performed to identify statistically significant biomarkers among groups with a score greater than 4. All analyses were performed using BMKCloud.^1^

### 2.8 Statistical analysis

All analyses were performed using SPSS statistical software 26.0 (IBM Corp., Armonk, NY, United States). A one-way ANOVA test was used for multiple comparisons, followed by Dunnett’s post-hoc test to assess statistical significance between groups. The results were represented by mean ± Standard Error of Means (SEM). Differences were considered statistically significant at *p* < 0.05. Histograms were plotted using GraphPad Prism 8.0 software (GraphPad Inc., San Diego, CA, United States).

## 3 Results

### 3.1 Growth performance

There was no significant difference (*p* < 0.05) in the mortality of broilers in any phase among groups (data not shown). Initial body weight (BW) of broilers among the groups exhibited no significant difference (*p* > 0.05, Table 3). However, the BW of broilers at 28 days of age and BWG aged 1–28 days significantly increased (*p* < 0.05) in MGUE group compared to the CON group and MOL group, while having no significant difference (*p* > 0.05) in the FI and F/G among groups. Compared to the CON group and MGUE group, the MOL group exhibited a significant decrease in BW of broilers at 56 and 86 days of age, as well as in BWG and FI aged 1–56 and 1–84 days, along with a significant increase in FCR (F/G) aged 1–84 days. Additionally, there was no difference in these parameters between the CON group and MGUE group.

**Table 3:** Effect of DON and ZEN contamination and GUE on the growth performance of broilers.

Items	CON	MOL	MGUE	SEM	*p*-value
Initial BW, kg	0.040	0.041	0.040	0.001	0.858
d 1–28					
BW (28 d), kg	0.547^b^	0.540^b^	0.564^a^	0.011	0.047
BWG, kg	0.503^b^	0.501^b^	0.521^a^	0.009	0.048
FI, kg	1.342	1.282	1.344	0.053	0.056
F/G	2.647	2.555	2.565	0.118	0.962
d 1–56					
BW (56 d), kg	1.678^a^	1.515^b^	1.703^a^	0.025	0.001
BWG, kg	1.637^a^	1.474^b^	1.663^a^	0.025	0.001
FI, kg	5.085^a^	4.875^b^	5.157^a^	0.076	0.044
F/G	3.104	3.317	3.105	0.074	0.095
d 1–84					
BW (84 d), kg	3.442^a^	3.046^b^	3.500^a^	0.041	0.001
BWG, kg	3.402^a^	3.006^b^	3.460^a^	0.039	0.001
FI, kg	10.970^b^	10.615^b^	11.040^a^	0.184	0.034
F/G	3.225^b^	3.531^a^	3.192^b^	0.050	0.001

BW, body weight; BWG, BW gain; FI, feed intake; FCR (F/G), feed/gain; SEM, standard error of means. Values with the same or no letter superscripts in the same row mean no significant difference (*p* > 0.05), while with different letter superscripts mean significant difference (*p* < 0.05).

### 3.2 Intestinal morphology

**Figure 1:**
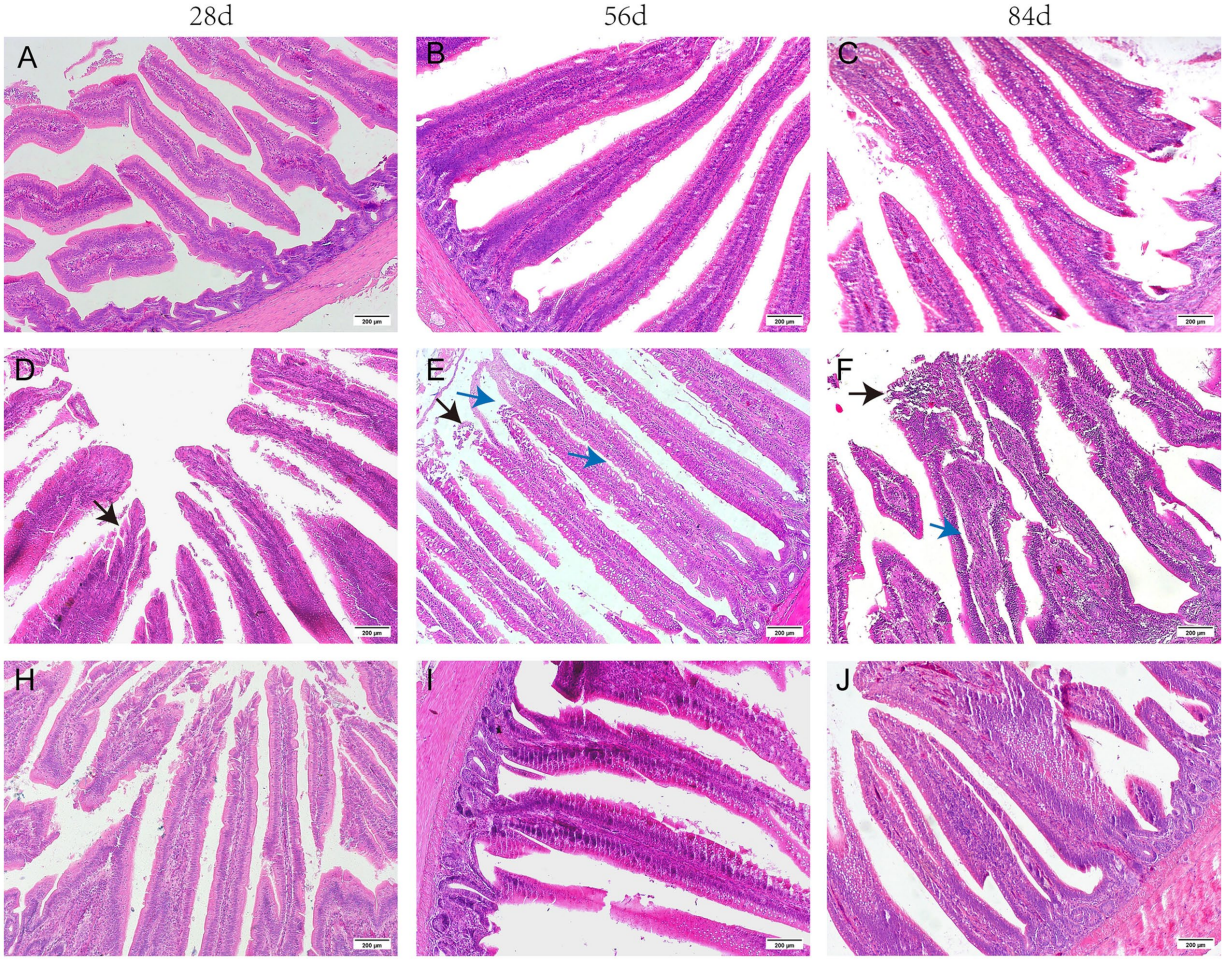
The histomorphology of the H&E stained jejunum in broilers. **(A–C)** CON group; **(D–F)** MOL group; **(H–J)** MGUE group. Black arrow: the shed epithelial cells of villi. Blue arrow: the Lamina propria edema of villi. Scale bar represents 200 μm.

As illustrated in Figure 1, the jejunum was well-formed, displaying a clear arrangement of villi and regular crypts in broilers from the CON group. In contrast, broken villi, swollen tips, and shed epithelial cells were noted in the jejunum of broilers at 28, 56, and 84 days of age in the MOL group. When fed a diet supplemented with GUE, the jejunum of broilers at 28 days of age showed reduced detachment of the epithelial cells, along with normal villi and abundant lymphocytes in the lamina propria at 56 and 84 days of age.

The morphology in the jejunum of broiler chickens was shown in Table 4. In comparison to the CON group, both the villus height and the ratio of villus height to crypt depth (V/C) significantly decreased (*p* < 0.05) in the MOL group. Compared to the MOL group, the MGUE group exhibited an increased villus height and V/C ratio at all ages (*p* < 0.05). Additionally, the villus height at 56 and 84 days of age, as well as V/C ratio at 84 days of age, significantly increased (*p* < 0.05) in the MGUE group compared to the CON group.

**Table 4:** Effect of DON and ZEN contamination and GUE on jejunum morphology in broilers.

Items	CON	MOL	MGUE	SEM	*p*-value
28d					
Villus height/μm	794.54^a^	753.14^b^	786.52^a^	23.17	0.039
Crypt depth/μm	165.66	171.93	159.53	11.26	0.092
Villus height /crypt depth	4.80^a^	4.38^b^	4.93^a^	0.27	0.025
56d					
Villus height/μm	925.20^b^	895.50^c^	958.41^a^	7.33	0.027
Crypt depth/μm	188.37	193.4	189.50	1.94	0.453
Villus height /crypt depth	4.91^a^	4.63^b^	5.05^a^	0.48	0.029
84d					
Villus height/μm	1109.75^b^	1059.23^c^	1253.28^a^	28.75	0.019
Crypt depth/μm	208.57	209.46	213.52	10.67	0.069
Villus height /crypt depth	5.32^b^	5.06^c^	5.87^a^	0.51	0.023

SEM, standard error of means; Values with the same or no letter superscripts in the same row mean no significant difference (*p* > 0.05), while with different letter superscripts mean significant difference (*p* < 0.05).

### 3.3 Intestinal antioxidant parameters

The antioxidant indices in jejunum mucosa of broiler chickens were presented in Table 5. Compared to the CON group, the MOL group exhibited significantly decreased activities of SOD and GSH-Px and a significantly increased content of MDA (*p* < 0.05) in the jejunal mucosa of broilers at 28, 56 and 84 days of age. In the MGUE group, compared to the MOL group, SOD activity significantly increased (*p* < 0.05) at 28 and 56 days of age, and GSH-Px activity significantly increased (*p* < 0.05) at 56 and 84 days of age, while MDA content decreased (*p* < 0.05) at 28, 56, and 84 days of age. However, the MGUE group showed significantly lower GSH-Px activity at 28 days of age and SOD activity at 84 days of age, along with a higher MDA content at 56 and 84 days of age compared to the CON group (*p* < 0.05). These results indicated that the MOL diet impaired the antioxidant function in the jejunal mucosa of broilers, and the addition of GUE could mitigate this negative impact.

**Table 5:** Effect of DON and ZEN contamination and GUE on antioxidant indices in broilers.

Items	CON	MOL	MGUE	SEM	*p*-value
28d					
MDA, nmol/g	13.13^b^	15.12^a^	12.15^b^	1.50	0.029
GSH-Px, nmol/min/g	157.19^a^	130.02^b^	137.94^b^	11.80	0.035
SOD, U/g	147.61^a^	120.29^b^	150.32^a^	10.26	0.031
56d					
MDA, nmol/g	14.40^c^	16.93^a^	15.18^b^	1.06	0.025
GSH-Px, nmol/min/g	298.59^a^	245.97^b^	292.91^a^	15.91	0.013
SOD, U/g	231.50^b^	226.82^c^	245.51^a^	10.48	0.045
84d					
MDA, nmol/g	15.74^c^	20.27^a^	17.82^b^	0.84	0.032
GSH-Px, nmol/min/g	334.04^a^	292.50^b^	342.00^a^	12.52	0.014
SOD, U/g	237.62^a^	206.41^b^	208.39^b^	12.88	0.001

SOD, superoxide dismutase; GSH-Px, glutathione peroxidase enzyme; MDA, malondialdehyde; SEM, standard error of means. Values with the same or no letter superscripts in the same row mean no significant difference (*p* > 0.05), while with different letter superscripts mean significant difference (*p* < 0.05).

### 3.4 Gut barrier-related genes and pro-inflammatory gene expression

**Figure 2:**
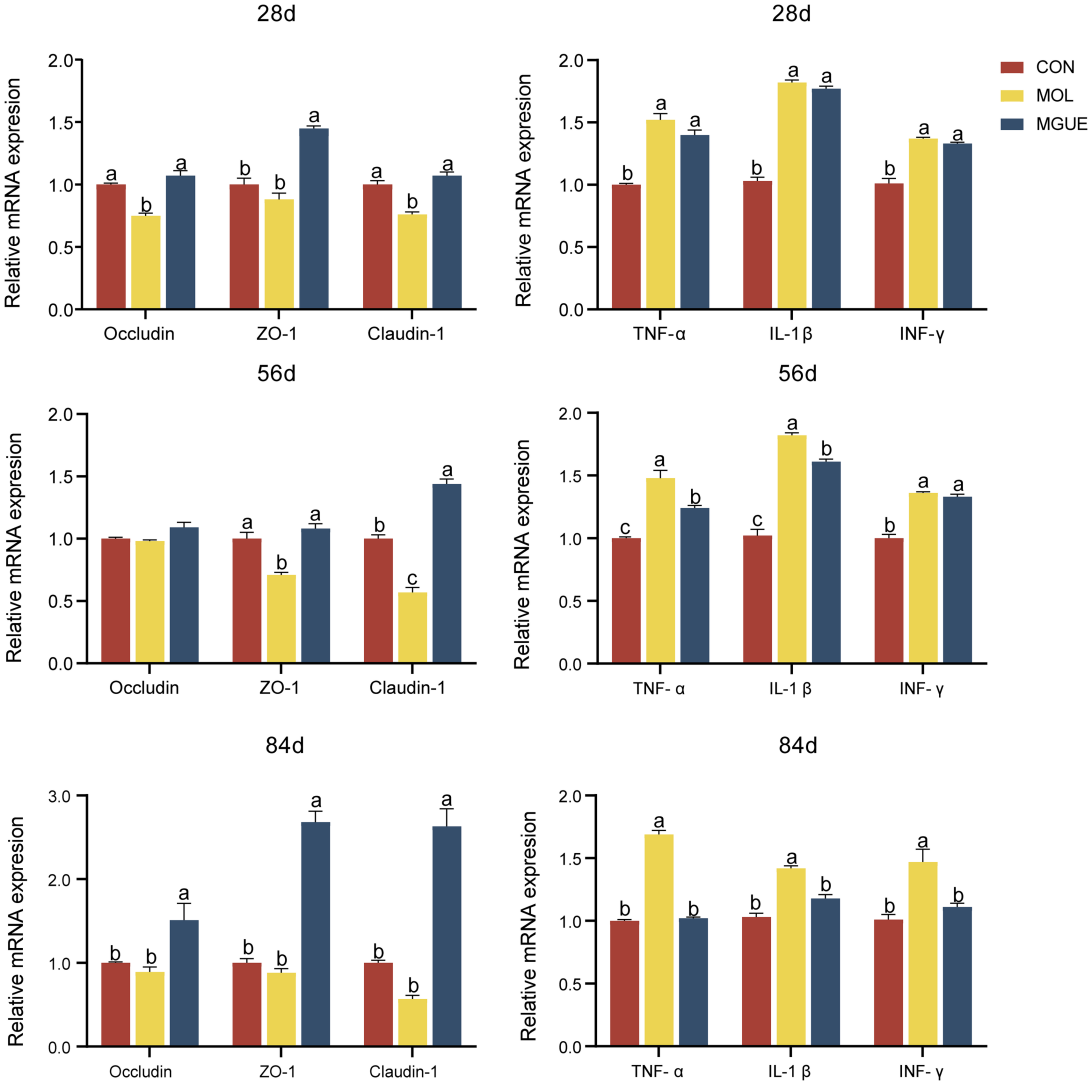
The expression of the gut barrier-related genes and pro-inflammatory genes in the jejunal mucosa of broilers. Values with the same or no letter superscripts mean no significant difference (*p* > 0.05), while with different letter superscripts mean significant difference (*p* < 0.05).

The mRNA expression of the gut barrier-related genes and pro-inflammatory genes in the jejunal mucosa of broilers were shown in Figure 2. Compared to the CON group, the MOL group exhibited a significantly reduced expression of *Occludin* and *Claudin-1* in the jejunal mucosa of broiler at 28 days of age, as well as a decreased (*p* < 0.05) expression of *Claudin-1* and *ZO-1* in broiler at 56 days of age, while there was no difference (*p* > 0.05) in the expression of these genes in broilers at 84 days of age. The expression levels of *Occludin*, *ZO-1*, and *Claudin-1* in broilers at 28 and 84 days of age, as well as *ZO-1* and *Claudin-1* in broilers at 56 days of age, were significantly elevated (*p* < 0.05) in the MGUE group compared to the MOL group. Additionally, broilers in the MGUE group exhibited increased (*p* < 0.05) expression levels of *Occludin*, *ZO-1*, and *Claudin-1* at 84 days of age, as well as *ZO-1* at 28 days and *Claudin-1* at 56 days compared to the CON group.

Compared to the CON group, the expression of *IL-1β*, *TNF-α*, and *INF-γ* significantly increased (*p* < 0.05) in the jejunal mucosa of broilers in the MOL group at 28, 56, and 84 days of age. The expression of *TNF-α* and *IL-1β* in broilers at 56 days of age, and *IL-1β*, *TNF-α*, and *INF-γ* in broilers at 84 days, were significantly lower (*p* < 0.05) in the MGUE group compared to the MOL group. Additionally, the expression of *IL-1β*, *TNF-α*, and *INF-γ* in broilers at 28 and 56 days of age was significantly higher (*p* < 0.05), while no significant differences (*p* > 0.05) were observed in broilers at 84 days of age in the MGUE group compared to the CON group.

### 3.5 Cecal microbiota

**Figure 3:**
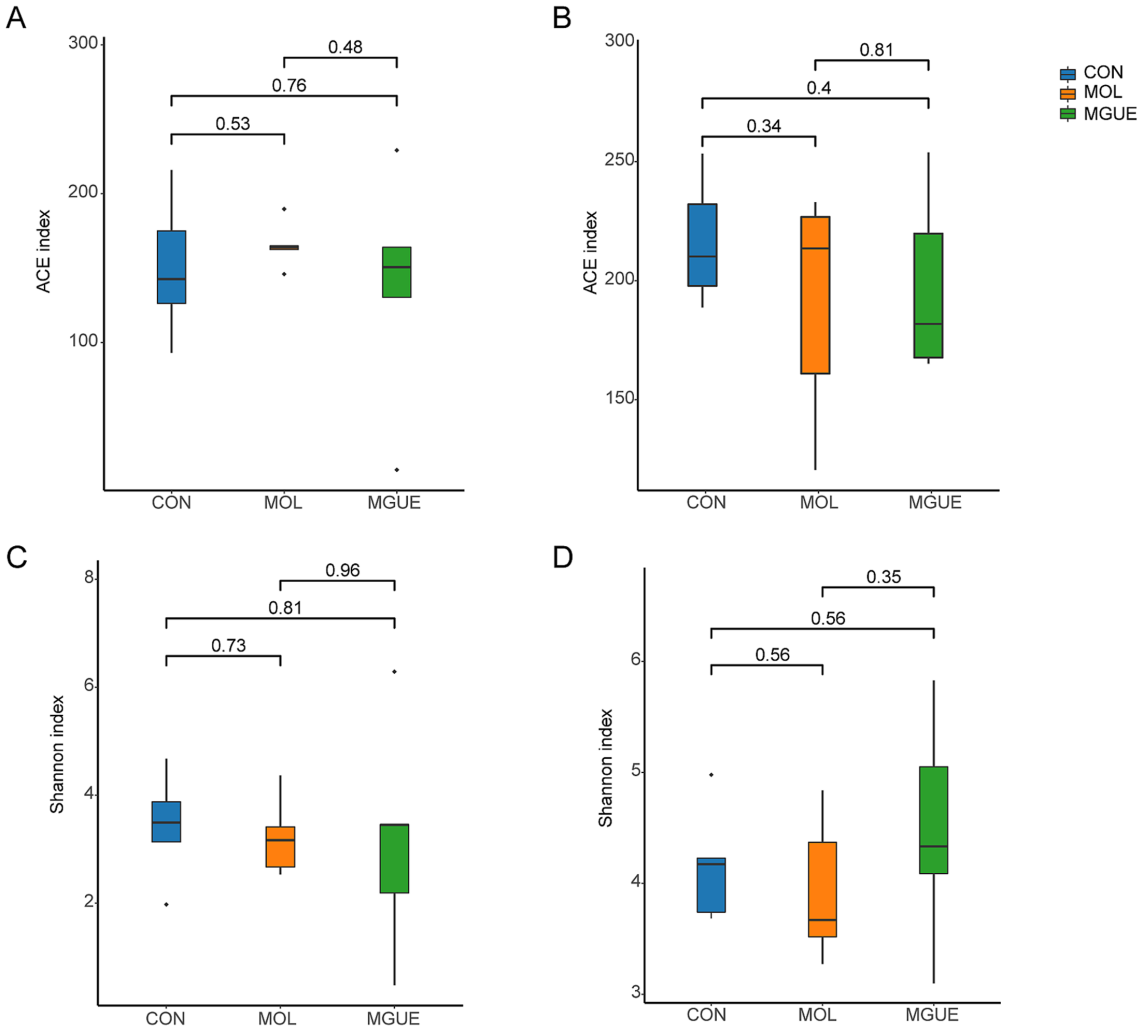
Diversity indices of the cecal microbiota of broilers. **(A)** ACE index of cecum of 28-day-old broilers. **(B)** ACE index of the cecum of 84-day-old broilers. **(C)** Shannon index of the cecum of 28-day-old broilers. **(D)** Shannon index of cecum of 84-day-old broilers.

The Alpha diversity of the cecal microbiota was assessed using the ACE and Shannon indices. Results indicated that there were no significant differences (*p* > 0.05) in the ACE and Shannon indices in broilers at 28 and 84 days of age among the groups (Figure 3).

**Figure 4:**
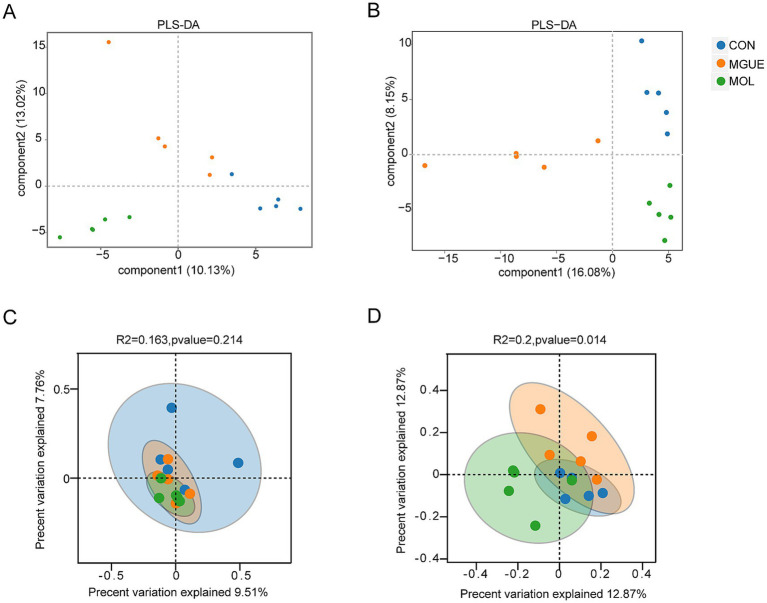
The Beta diversity of the cecal microbiota of broilers. **(A)** PLS-DA of the cecum of 28-day-old broilers. **(B)** PLS-DA of the cecum of 84-day-old broilers. **(C)** PCoA of the cecum of 28-day-old broilers. **(D)** PCoA of the cecum of 84-day-old broilers.

Beta diversity was visually analyzed by plotting the distances between samples using Partial Least Squares Discriminant Analysis (PLS-DA) and Principal Coordinates Analysis (PCoA). The PLS-DA and PCoA analyses demonstrated that at 28 days of age, there was a significant separation in the cecal microbial community between the MOL and CON groups; however, no complete separation was noted between the MOL and MGUE groups (Figures 4A,C). At 84 days of age, a distinct separation among the three groups was observed, with samples clustering within each group (Figures 4B,D).

**Figure 5:**
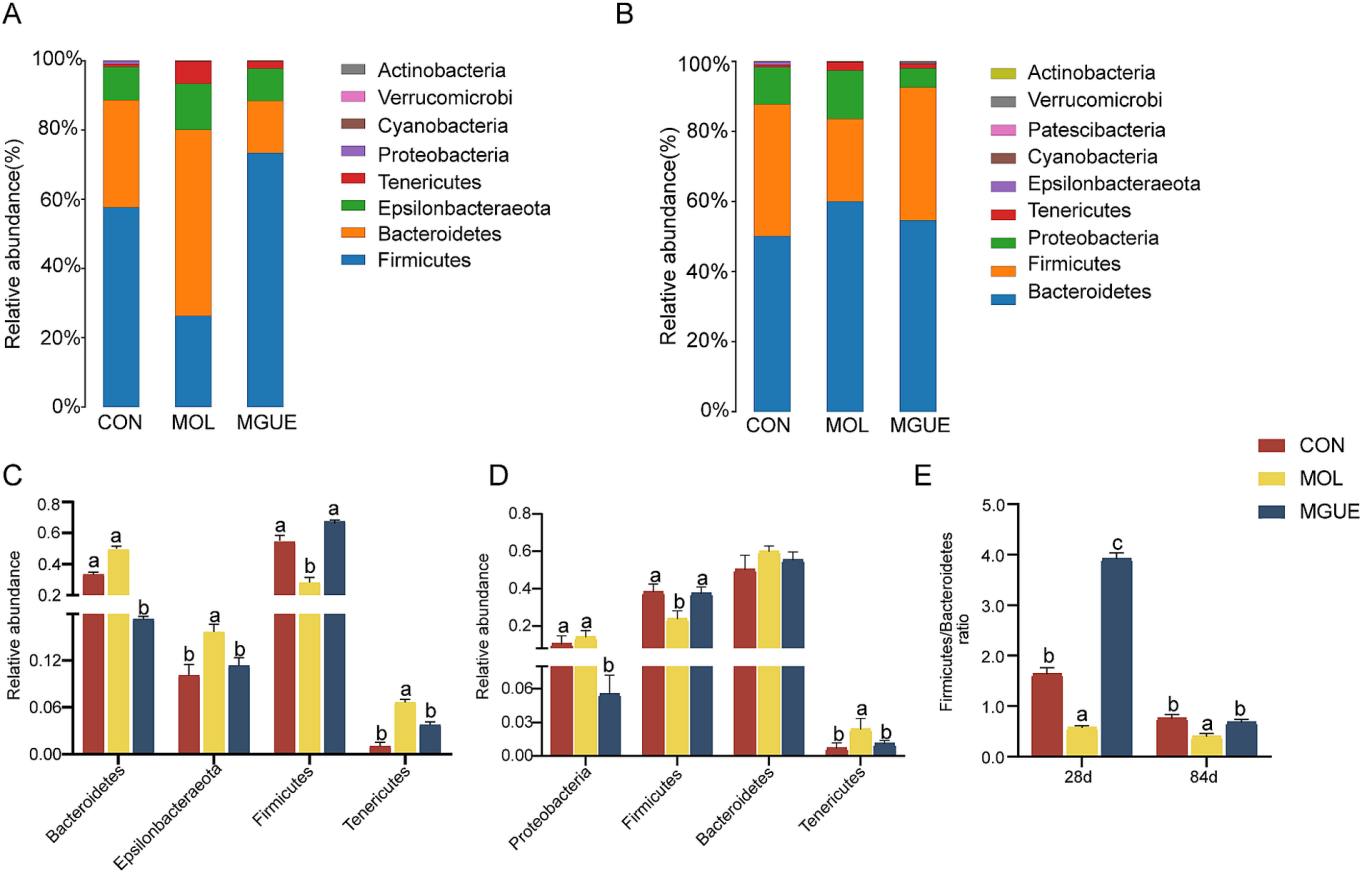
Relative abundance of cecal microbiota in broiler chickens at the phylum level. **(A,B)** Relative abundance taxa at 28 and 84 d of age. **(C,D)** Relative abundance difference analysis of cecal bacterial species at the phylum level at 28 and 84 d of age, respectively. **(E)** *Firmicutes*/*Bacteroidetes* (F/B) ratio at 28 and 84 d of age. Values with the same letter superscripts mean no significant difference (*p* > 0.05), while with different letter superscripts mean significant difference (*p* < 0.05).

Ten bacterial phyla were identified in cecum microbiota of broiler chickens from three groups (Figure 5). At 28 days of age, the most predominant bacterial phyla included *Firmicutes*, *Bacteroidetes*, *Epsilonbacteraeota* and *Tenericutes* across all three groups (Figure 5A). Compared to the CON group, the relative abundance of *Epsilonbacteraeota* and *Tenericutes* significantly increased, while that of *Firmicutes* decreased in the MOL group, resulting in a decreased *Firmicutes* / *Bacteroidetes* (F/B) ratio (*p* < 0.05); the relative abundance of *Bacteroidetes* decreased (*p* < 0.05), with no significant difference in *Firmicutes* and *Epsilonbacteraeota* (*p* > 0.05) in the MGUE group. Additionally, the relative abundance of *Firmicutes* was significantly higher, whereas that of *Bacteroidetes* and *Tenericutes* was significantly lower (*p* < 0.05), resulting in an increased F/B ratio in the MGUE group compared to the MOL group (Figures 5C,E).

At 84 days of age, *Bacteroidetes* was the most predominant phylum, followed by *Firmicutes, Proteobacteria*, and *Tenericutes* (Figure 5B). Compared to the CON group, the relative abundance of *Tenericutes* significantly increased, and that of *Firmicutes* and the F/B ratio significantly decreased (*p* < 0.05) in the MOL group; the relative abundance of *Proteobacteria* significantly decreased (*p* < 0.05), while no significant difference (*p* > 0.05) was observed in the abundance of *Firmicutes*, *Bacteroidetes* and *Tenericutes* in the MGUE group. However, the relative abundance of *Proteobacteria* and *Tenericutes* significantly decreased, while that of *Firmicutes* and the F/B ratio significantly increased (*p* < 0.05) in the MGUE group compared to the MOL group (Figures 5D,E).

**Figure 6:**
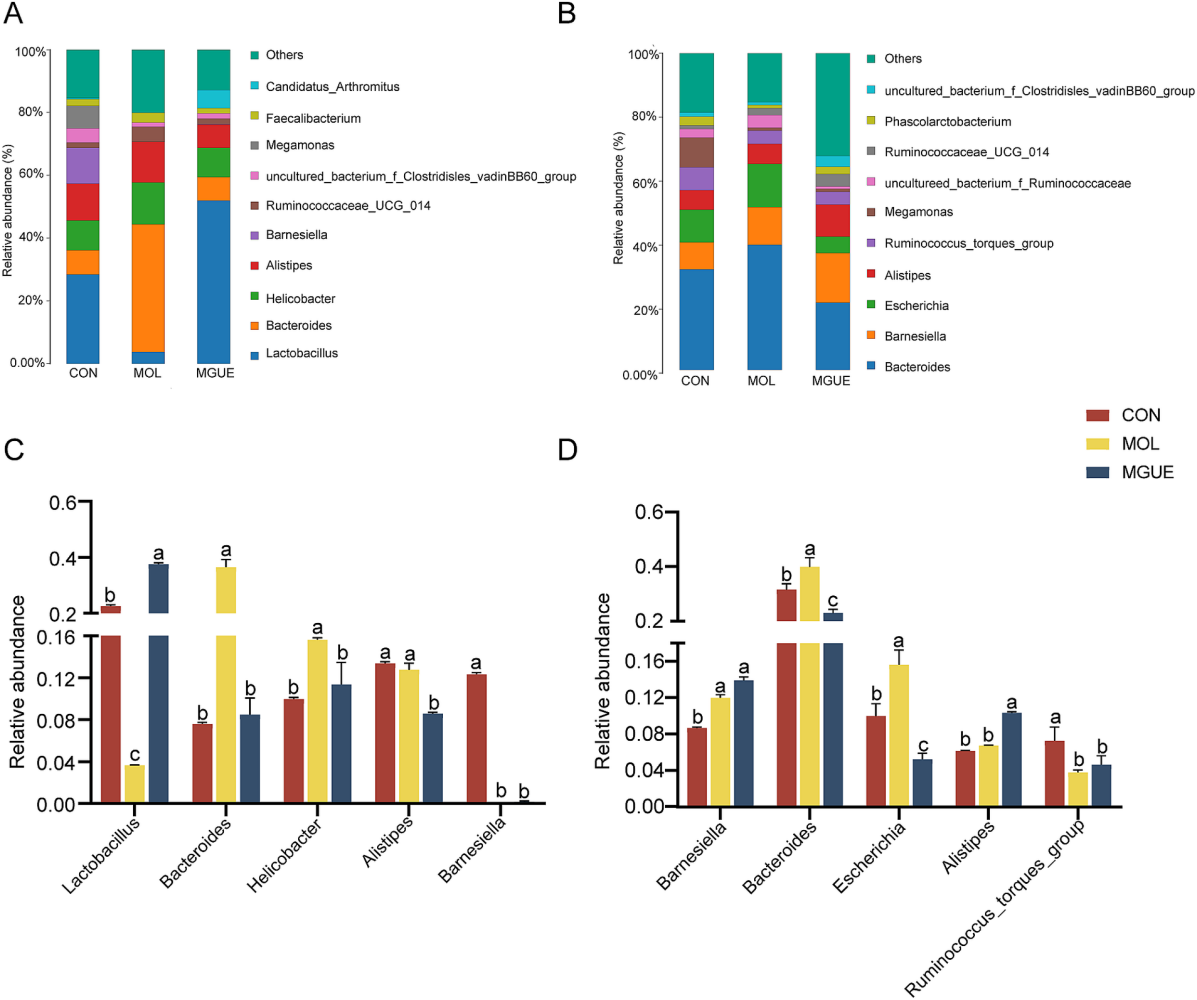
Relative abundance of cecal microbiota in broiler chickens at the genus level. **(A,B)** Relative abundance taxa at 28 and 84 d of age. **(C,D)** Relative abundance difference analysis of cecal bacterial species at the genus level at 28 and 84 d of age, respectively. Values with the same letter superscripts mean no significant difference (*p* > 0.05), while with different letter superscripts mean significant difference (*p* < 0.05).

The top ten genera by relative abundance in the cecal microbiota were presented in Figure 6. At 28 days of age, *Lactobacillus*, *Bacteroides*, *Helicobacter*, *Alistipes*, and *Barnesiella* were the predominant genera in the cecal microbiota of chickens (Figure 6A). The relative abundance of *Lactobacillus* and *Barnesiella* significantly decreased, while that of *Bacteroides* and *Helicobacter* significantly increased (*p* < 0.05) in the cecal microbiota in the MOL group compared to the CON group. The relative abundance of *Alistipes, Bacteroides* and *Helicobacter* significantly decreased (*p* < 0.05), while that of *Lactobacillus* significantly increased (*p* < 0.05) in the MGUE group compared to the MOL group. Additionally, the relative abundance of *Lactobacillus* significantly increased, while that of *Alistipes* and *Barnesiella* significantly decreased (*p* < 0.05) in the MGUE group compared to the CON group (Figure 6C).

At 84 days of age, *Bacteroides, Barnesiella, Escherichia, Alitipes*, and *Ruminococcaceae_torques_group* were the predominate genera (Figure 6B). The relative abundance of *Barnesiella*, *Bacteroides* and *Escherichia* significantly increased (*p* < 0.05), while that of *Ruminococcaceae_torques_group* significantly decreased (*p* < 0.05) in the MOL group. The relative abundance of *Escherichia* and *Bacteroides* significantly decreased (*p* < 0.05), and that of *Alistipes* significantly increased (*p* < 0.05) in the MGUE group compared to the MOL group. Additionally, the relative abundance of *Barnesiella* and *Alitipes* was significantly higher, while that of *Bacteroides*, *Escherichia* and *Ruminococcaceae_torques_group* was lower (*p* < 0.05) in the MGUE group compared to the CON group (Figure 6D).

**Figure 7:**
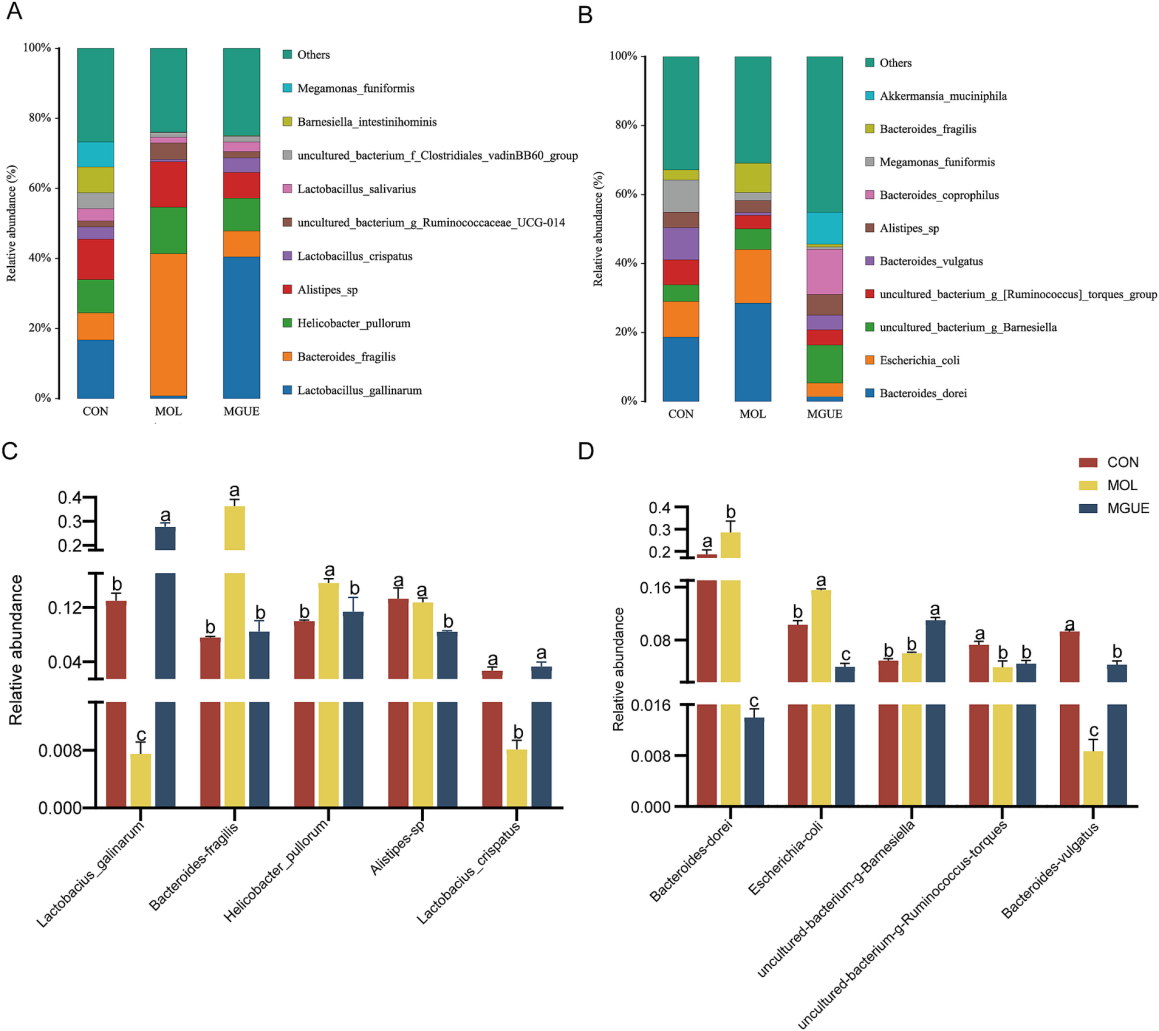
Relative abundance of gut bacterial composition at the species level. **(A,B)** Relative abundance taxa at 28 and 84 d of age. **(C,D)** Relative abundance difference analysis of cecal microbiota at the species level at 28 and 84 d of age, respectively.

The top ten species by relative abundance in the cecal microbiota were presented in Figure 7. At 28 days of age, the most abundant taxa in three groups were *Alistipes_sp., Bacteroides_fragilis*, *Helicobacter_pullorum*, *Lactobacillus_crispatus* and *Lactobacillus_gallinarum* (Figure 7A). Compared to the CON group, the relative abundance of *Helicobacter_pullorum* and *Bacteroides_fragilis* significantly increased (*p* < 0.05), while that of *Lactobacillus_gallinarum* and *Lactobacillus_crispatus* significantly decreased in the MOL group. Additionally, the MGUE group showed a significant decrease (*p* < 0.05) in the relative abundance of *Alistipes_sp., Helicobacter_pullorum* and *Bacteroides_fragilis,* and an increase in *Lactobacillus_gallinarum* and *Lactobacillus_crispatus* compared to the MOL group (Figure 7C). Compared to the CON group, the relative abundance of *Alistipes_sp* significantly decreased, and that of *Lactobacillus_gallinarum* increased (*p* < 0.05), while there was no significant difference (*p* > 0.05) in that of the other three species in the MGUE group.

At 84 days of age, *Bacteroides_vulgatus*, *Bacteroides_dorei*, *Escherichia_coli*, *uncultured_bacterium_g_Barnesiella*, and *uncultured_bacterium_g*_[*Ruminococcus*]*_torques_group* were the dominant species in all groups (Figure 7B). The relative abundance of *Bacteroides_dorei* and *Escherichia_coli* significantly increased (*p* < 0.05), while that of *Bacteroides_vulgatus* and *uncultured_bacterium_g*_[*Ruminococcus*]*_torques_group* significantly decreased (*p* < 0.05) in the MOL group compared to the CON group (*p* < 0.05). In the MGUE group, the relative abundance of *Bacteroides_dorei* and *Escherichia_coli* significantly decreased compared to both the CON and MOL groups, while that of *Bacteroides_vulgatus* and *uncultured_bacterium_g_Barnesiella* significantly increased (*p* < 0.05) compared to both the MOL group. Additionally, the abundance of *uncultured_bacterium_g*_[*Ruminococcus*]*_torques_group* and *Bacteroides_vulgatus* was significantly lower (*p* < 0.05) in the MGUE group compared to the CON group (Figure 7D).

**Figure 8:**
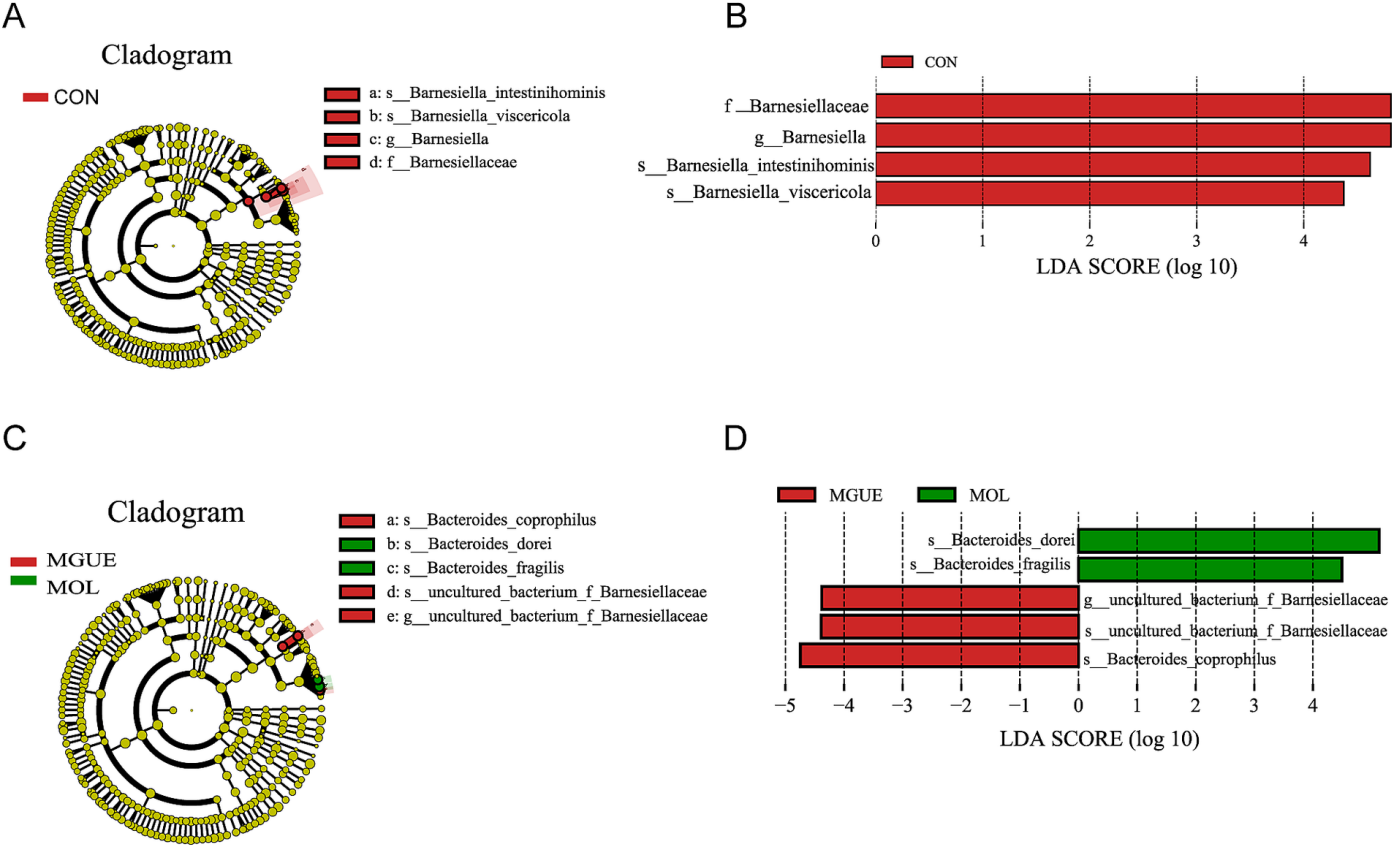
LEfSe taxonomic cladogram and LDA score of cecal microbiota. **(A,B)** LEfSe taxonomic cladogram and LDA score at 28 days of age. **(C,D)** LEfSe taxonomic cladogram and LDA score at 84 days of age. The circles radiating from the center to the outer edges of the evolutionary branch map represent classification levels from phylum to species, with the circle’s diameter proportional to the taxon’s abundance. The yellow nodes represent taxonomic units that exhibit no significant differences between groups. The LDA histograms display the LDA scores, and the difference is significant when LDA >4. f, family; g, genus; s, species.

The Linear Discriminant Analysis (LDA) combined with LDA Effect Size (LEfSe) analysis was employed to further examine the changes in the cecum microbiota at both the genus and species levels in broilers (LDA scores >4). At 28 days of age, *f_Barnesiellaceae*, *g_Barnesiella*, *s_Barnesiella_visceriricola* and *s_Barnesiella_intestinihominis* were significantly enriched in the CON group (Figures 8A,B); however, no biomarkers were significantly enriched in the MOL and MGUE groups. At 84 days of age, *s_Bacteroides_dorei* and *s_Bacteroides_fragilis* were more abundant in the MOL group, whereas the *s_Bacteroides_coprophilus*, *g_uncultured_bacterium_f_Barnesiellaceae* and *s_uncultured_bacterium_f_Barnesiellaceae* was overrepresented in the MGUE group (Figures 8C,D).

## 4 Discussion

### 4.1 Growth performance

Many studies have demonstrated that mycotoxin contamination reduced the nutritional value of feedstuff and the growth performance of animals, and caused health problems such as inflammation and reduced antioxidant capacity (35). A low-level exposure to mycotoxins resulted in various metabolic disturbances, leading to decreased productivity in animals (36). Poultry exhibited a comparatively higher tolerance to ZEN, and a diet containing 2.0 mg ZEN/kg had no impact on broilers aged 1 to 21 days, but reduced weight gain and feed conversion efficiency in broilers throughout the entire experimental period aged 1 to 42 days (37). Diet contaminated with 1 or 5 mg DON/kg feed did not affect performance or the absolute and relative organ weights, but significantly altered the intestinal morphology with a shorter villi and a reduced villus surface area in broilers (38). Exposure to 20, 5, and 0.5 mg/kg of FB, DON, and ZEN, respectively, did not impact the performance, biochemistry, or histopathology in ducks, but ducks exposed to the combination of these mycotoxins exhibited reduced body weight gain and feed conversion ratio, as well as an increased liver sphingosine to sphingosine-1-phosphate ratio, during a 12-day study (39). The research by Tardieu et al. (40) indicated that although previous studies found no toxicity of ZEN and FB1 in the livers of turkeys and chickens, exposure to 7.5 and 0.6 mg/kg of FB and ZEN led to *α*-zearalanol and *β*-zearalenol persisting in the livers of turkeys at low concentrations for a long time, but not in chickens. GUE, which contains a variety of bioactive components, enhanced the development performance by promoting antioxidant and anti-inflammatory responses, and improving intestinal health in poultry when utilized as feed additives (32, 41). Numerous researchers have concluded that licorice powder or GUE supplementation significantly improved FCR, BW and cumulative BWG in broilers (42, 43). In this study, the diet containing a low dose of DON and ZEN reduced BW of broilers at 56 and 84 days of age, BWG and FI aged 1–56 and 1–84 days, and increased F/G aged 1–84 days, while there was no impact on broilers aged 1 to 28 days. Additionally, the supplementary GUE in the MOL diet significantly improved these decreased parameters caused by DON and ZEN contamination, and returned to a normal level as same as the CON group. This finding indicated that long-term intake of low-dose DON and ZEN reduced the growth performance and feed conversion rate in broilers, with this adverse effect being cumulative. However, GUE as feed additives could effectively mitigate the damage to the growth performance of broilers caused by low-dose DON and ZEN contamination.

It should be noted that after chronic feeding broilers with low-dose DON and ZEN for 84 days, we did not detect the presence of DON and ZEN in the liver and breast muscle, which aligns with previous studies on DON (38). Riahi et al. (44) analyzed the concentrations of DON in broilers fed DON at 5 or 15 mg/kg for 42 days and found that DON was below the limit of quantification (5 ng/mL) in plasma and liver but was detected in excreta. This result was controlled by gut and liver enzymes, which facilitate the oxidation, reduction, hydrolysis, and/or conjugation of toxins (45). The detection of DON in excreta may be attributed to the rapid clearance of this toxin into excreta (44).

### 4.2 Intestinal morphology and antioxidant

The duodenum and proximal jejunum were primarily sites for nutrient digestion and absorption in poultry (46). The intestinal morphology is closely associated with the absorption of nutrients and growth performance in animals. The VH, CD, and V/C ratio profoundly affect nutrient absorption, and are frequently used as indicators for assessing the physiological function and injury degree of intestinal tissue (47, 48). The height of villus indicated absorption surface, whereas the V/C ratio reflected intestinal functions (49). Longer villi and elevated V/C ratios provide a greater surface area for nutrient absorption, promoting a healthy intestinal tract (50). Wageha et al. (38) found that both 1 and 5 mg/kg of DON in the diet significantly altered small intestinal morphology, with the VH being notably shorter in the jejunum of broiler chickens. The intestinal villi were swollen, ulcerated, and fractured when broilers were fed DON-contaminated feed (51). Similarly, we observed a marked decrease in VH and V/C ratio, with visible villi that were broken, swollen tips, and shed epithelial cells in the jejunum of broilers fed a diet contaminated with DON and ZEN. However, after the MOL diet was supplemented with GUE, the VH and V/C ratio significantly increased and returned to normal levels in the jejunum of broilers, along with a normal morphology of the jejunum. The effect of GUE and its bioactive components on intestinal morphology has been validated by several studies (52, 53). Dietary GUE increased intestinal villus length and V/C ratio of broilers (41). These results indicated that the low-dose ZEN and DON impaired intestinal health and nutrient absorption; however, GUE could protect against the damage caused by DON and ZEN.

Antioxidant system plays an important role in the health and growth of animals. The SOD and GSH-Px are vital components of the antioxidant enzyme system, effectively scavenging free radicals and preserving the intracellular redox balance. MDA, the end product of lipid peroxidation, indicates the degree of oxidative damage within the body. Several studies have shown that broilers exposed to DON exhibited a decrease in jejunal CAT and GSH-Px activities, resulting in oxidative stress and an imbalance of redox status (17). ZEN reduced SOD and GSH-Px activities while increasing MDA content, leading to oxidative stress in broilers (54). In this study, the diet contaminated with DON and ZEN significantly decreased the activities of SOD and GSH-Px in the jejunum mucosa of broilers, while increasing the content of MDA. This demonstrated that prolonged intake of low doses of DON and ZEN impaired the antioxidant function of broilers. Moreover, the additional GUE in the MOL diet significantly increased the activities of SOD and GSH-Px while reducing MDA content in the jejunum mucosa of broilers. However, the GSH-Px activity at 28 days of age and the SOD activity at 84 days of age were significantly lower, while the MDA level was higher at 56 and 84 days of age compared to the normal levels observed in the control broilers. Dietary supplementation with GUE has been shown to eliminate reactive oxygen species (ROS) and improve overall and intestinal health in animals (55, 56). The supplementary GUE increased the activity of SOD and GSH-Px, and reduced MDA content in the jejunum mucosa of broilers (32). These results suggested that GUE could mitigate the damage caused by the long-term intake of low-dose DON and ZEN on the antioxidant function of the gut in broilers.

### 4.3 Intestinal barrier and inflammation

The integrity of the intestinal epithelium acts as a physical barrier against enteric pathogen invasion and ensures optimal nutrient absorption (57). Epithelial cells are connected by tight junction (TJ) complexes consisting of TJ proteins to maintain the integrity of the intestinal epithelial barrier (58). Tight junctions are crucial structures in epithelial and endothelial cells that help regulate the permeability of the paracellular pathway (the space between adjacent cells) and maintain cell polarity. The claudins, occludin and zonula occludens (ZO) are the main TJ proteins, with Claudins serving as crucial determinants of the permeability of the space between two adjacent cells (59). Claudin-1, a key constituent of the TJ complex, maintains the integrity of the paracellular barrier and regulates water homeostasis. The presence of ZO-1 and occludin alone in the tight junctions was not sufficient to achieve a paracellular seal in intestinal epithelial cells when claudin was specifically removed from intercellular junctional complexes (60). The intestinal epithelium was also the first barrier against food contaminants and was highly sensitive to *Fusarium* toxins, including DON and ZEN (15). AFB1-contaminated diet significantly decreased the expression of *claudin*-1 and *occludin* in jejunum of broiler chickens (61). Mycotoxins could specifically target claudin and directly increased intestinal barrier permeability, resulting in increased bacterial translocation (62).

Inflammatory cytokines are signaling molecules released by various cells, playing key roles in the regulation of immune responses and inflammation. Tumor necrosis factor-*α* (TNF-α) is known to induce apoptosis and inflammatory responses in intestinal epithelial cells (63). Cytokine INF-*γ* promoted the maturation of cytotoxic T lymphocytes, B cell proliferation, and antibody production (64), and induced endocytosis of tight junction proteins (65). IL-1β promoted inflammation by stimulating the production of other cytokines, recruiting additional immune cells, and enhancing the expression of adhesion molecules on endothelial cells (66). Feeding a diet containing 1007.5 μg/kg DON and 265.4 μg/kg ZEN for 3 weeks led to a significant increase in the expression of *TNF-α*, *IL-1β*, *IFN-γ*, and *IL-6* in the jejunum of piglets (67). The addition of 5 mg/kg DON (44) or both 1.5 mg DON/kg and 20 mg FB/kg (68) to the diet increased the expression of proinflammatory cytokines in in the jejunum of broilers, such as *IL-6*, *IFN-γ*, and *IL-1β*, suggesting that DON at these levels was immunostimulatory and proinflammatory. In this study, we observed that the inclusion of DON and ZEN in the diet decreased the expression of *claudin-1*, *occludin* and *ZO-1*, while increasing the expression levels of pro-inflammatory cytokines *TNF-α*, *IL-1β* and *IFN-γ* in the jejunum of broilers. These results suggested that long-term intake of low dose DON and ZEN could impair intestinal barrier function and stimulate inflammatory responses, leading to damage in the intestinal health of broilers.

Previous studies have shown that GUE preserves intestinal integrity and mitigates the damage caused by challenges with *Campylobacter jejuni* by upregulating the expression of *occludin* and *JAM*, while downregulating inflammatory markers *IL-1β* and Toll-like receptors (*TLR*-4) in the jejunum of broilers (28). It has been well reviewed that GUE inhibits oxidative stress, enhances anti-inflammatory and antioxidant responses, and aids in the elimination of mycotoxins from the body (69). Murugan et al. (70) found that adding a flavonoid-rich extract of *Glycyrrhiza glabra* into the diet increased the expression of occludin and ZO-1 proteins in the colon of rats. Dietary supplementation of GUE improved growth performance and preserved intestinal integrity by upregulating the expression of *occludin* and *JAM*, while downregulating inflammatory markers *IL-1β* and Toll-like receptors (*TLR*-4) in the jejunum of broilers challenged with *C. jejuni*. In this study, the supplementary GUE in the contaminated diet increased the expression of *claudin-1*, *occludin*, and *ZO-1*, while decreasing the expression of *TNF-α*, *IFN-γ*, and *IL-1β* in the jejunum, although the expression of *IL-1β* did not return to a level comparable to that of the control group. These results suggested that GUE could mitigate the intestinal barrier dysfunction and inflammatory response induced by DON and ZEN contamination.

### 4.4 Variation in cecal microbiota diversity

Accumulating evidence demonstrates that the intestinal microbiota plays a key role in maintaining intestinal barrier integrity, energy metabolism, and immunity (71–73). There is a close interaction between gut microbiota and the mycotoxins ingested by animals. Although previous studies showed that DON intake decreased the richness and evenness of cecal microbiota (74), our study found that dietary contamination with a low dose of DON and ZEN had no significant effect on Alpha diversity of cecal microbiota based on the OTU level in 28- and 84-day-old broilers.

Beta diversity analysis primarily describes variations in composition among microbiota. In this study, both PLS-DA and PCoA analysis were employed to elucidate the discrepancy in cecal microbiota diversity. We observed that dietary DON and ZEN contamination significantly affected the structure of the cecal microbiota in broilers at 28 and 84 d of age. However, there was a distinct separation only in broilers at 84 d of age between the MOL and MGUE groups. Wu et al. (75) found that incorporating compound *Glycyrrhiza* polysaccharide into the diet influenced the structure of the cecal microbiota in broilers at 15 days of age. These results indicated that dietary DON and ZEN contamination significantly affected the composition of cecal microbiota in broilers, and it might take longer for GUE to restore the effect caused by the mycotoxins.

### 4.5 Variation in cecal microbiota composition

The species annotation results were analyzed to comprehend the mechanisms by which dietary DON and ZEN contamination and GUE supplementation, influenced growth and intestinal health through intestinal microbiota. In this study, *Firmicutes* and *Bacteroidetes* were the most predominant phyla, followed by *Tenericutes*, *Epslionbacteraeota*, or *Proteobacteria* in the cecum of broilers. *Firmicutes* and *Bacteroidetes* collectively influenced the host’s energy absorption and maintained intestinal health (76), and the *Firmicutes* to *Bacteroidetes* (F/B) ratio in the intestine influenced the host’s capacity to obtain energy from feed (77). A higher F/B ratio was frequently associated with improved growth performance (78). Our study found that the contamination of DON and ZEN led to a reduction in the relative abundance of *Firmicutes* and an increase in that of *Bacteroidetes*, resulting in a decreased F/B ratio in the cecum of broilers at 28 and 84 days of age. Consistent with this, Chang et al. (79) reported that ZEN contamination reduced weight gain by decreasing the intestinal F/B ratio in broilers. Additionally, the contamination of DON and ZEN increased the relative abundance of *Epsilonbacteraeota.* Research on *Epsilonbacteraeota* primarily focuses on its pathogenic members, which affect digestive health and various metabolic processes. Notable genera within this phylum included *Campylobacter* and *Helicobacter*, both of which were linked to gastrointestinal disease in humans and animals (75). These results indicated that the diet contaminated with a low-dose DON and ZEN could reduce the growth performance and impair intestinal health by influencing cecal microbiota composition in broilers.

It has been reported that dietary GUE exhibited antibacterial effects against *Pseudomonas aeruginosa*, *Shigella flexneri*, *Escherichia coli*, *Staphylococcus epidermidis*, and *S. aureus* (80). *Glycyrrhiza* polysaccharides significantly increased the F/B ratio in the cecum of broilers, which promoted microbial metabolic efficiency and subsequently improved host nutrient acquisition (81). In this study, we found that GUE significantly increased the abundance of *Firmicutes* and decreased that of *Bacteroidetes*, resulting in a greater F/B ratio in the cecum of broilers. Additionally, GUE supplementation decreased the relative abundance of *Epsilonbacteraeota* in the cecum of broilers at 28 d of age, and that of *Proteobacteria* at 84 d of age. *Proteobacteria* included opportunistic pathogens like *E. coli*, *Shigella*, *Salmonella*, and *Klebsiella* (82), which were associated with inflammatory responses and intestinal infectious diseases (83). These results suggested that GUE could reduce the negative impact of long-term intake of low-dose DON and ZEN on growth performance by improving the cecal microbiota composition in broilers.

Based on the analysis of cecal microbial composition, we found that dietary DON and ZEN contamination significantly affected the composition of cecal microbiota at the genus and species levels in broilers. Dietary DON and ZEN increased the relative abundance of *Bacteroides* (*Bacteroides fragilis*), *Helicobacter* (*Helicobacter pullorum*) at 28 days of age, and that of *Barnesiella*, *Bacteroides* (*Bacteroides dorei*) and *Escherichia* (*Escherichia coli*) at 84 days of age. *Helicobacter* and *Escherichia* easily colonized the intestines of humans and animals, causing various diseases by modulating the production of inflammatory factors and disrupting intestinal mucosal permeability, damaging the intestinal barrier (84–86). *Helicobacter_pullorum* (87) and *Escherichia_coli* (88) were common pathogenic bacteria found in the intestine, impacting nutrient absorption, immunity, and the self-repair functions of infected chickens. Chronic intake of low doses of DON and/or ZEN in naturally moldy diets impaired intestinal functions, leading to inflammation and disrupting the epithelial barrier by promoting *E. coli* proliferation in the intestines of piglets (67). *Helicobacter_pullorum* has been linked to gastrointestinal diseases in poultry, interacting with the host’s immune system and potentially triggering immune responses that affected gut health and disease susceptibility. The presence of *H. pullorum* in the intestines of poultry negatively impacted overall health, growth, and feed efficiency, all of which are crucial for the poultry industry (85).

*Bacteroides* are generally considered “friendly” commensals residing in the gut, involved in various metabolic activities of animals and maintaining intestinal homeostasis (89, 90). However, the composition of *Bacteroides* is diverse and complex, playing a crucial role in various metabolic activities in animals (90). While some members of *Bacteroides* are part of the normal GIT microbiota, they may cause opportunistic infections if the integrity of intestinal mucosal barrier is disrupted, causing diarrhea by producing enterotoxins on the surfaces of intestinal epithelial cells (91, 92). *Bacteroides* was positively correlated with serum inflammatory cytokines TNF-a, IL-1β, and IL-6 (31). *Bacteroides fragilisis* and *Bacteroides dorei* are acknowledged as opportunistic pathogen within the *Bacteroides genus. Bacteroides fragilis* adhered to the surfaces of intestinal epithelial cells and caused intestinal inflammatory response (93). DON and ZEN contamination increased the relative abundance of *Bacteroides*, including *Bacteroides*_*fragilis* and *Bacteroides*_*dorei*. LEfSe analysis also showed that both *Bacteroides*_*fragilis* and *Bacteroides*_*dorei* were biomarkers for the MOL group. These results suggested that the damage caused by dietary DON and ZEN to intestinal barrier function could increase the accumulation of *Bacteroides* and lead to a heightened risk of opportunistic infections.

In this study, we also observed that dietary DON and ZEN reduced the abundance of the genus *Lactobacillus* including *Lactobacillus_gallinarum* and *Lactobacillus_crispatus*, and *Barnesiella* in the cecum of broilers at 28 d of age, and that of *Bacteroides_vulgatus* and *Ruminococcaceae_torques_group* at 84 d of age. The abundance of *Barnesiella* was associated with short-chain fatty acids (SCFAs) production and the anti-inflammatory capability of broilers (94). Both *Bacteroides_vulgatus* and *Ruminococcaceae_torques_group* degrades carbohydrates to produce SCFAs and plays a role in the overall maintenance of gut health and the balance of microbial communities. The increased abundance of *Ruminococcaceae_torques_group* was associated with enhancing anti-inflammatory effects and bile acid metabolism, leading to an improvement in canine inflammatory bowel disease (IBD) (95). *Ruminococcaceae_torques_group* was positively correlated with the T helper (Th) 1/Th2 ratio and modulated gut immune responses in patients with type 2 diabetes (96). *B. vulgatus* ameliorated intestinal inflammation and relieved depressive symptoms through the gut–brain axis (97). *Lactobacillus* is a group of commensal bacteria known to modulate the immune function of the intestine and promote the health of the host. *Lactobacillus* quickly colonized the GIT of broilers after hatching, and their metabolic activity reduced the pH of the chyme, which helps prevent the growth of harmful intestinal bacteria (98). *L. gallinarum* is beneficial to intestinal health, modulating intestinal microbial composition, secreting protective metabolites (99), improving the intestinal absorption (100), and inhibiting the colonization of *Salmonella* in GIT (101). Thus, the diet containing DON and ZEN could inhibit the proliferation and colonization of beneficial microbiota in the intestines of broiler chickens, particularly during the early stages of growth. These results suggested that dietary DON and ZEN could disrupt the balance of gut microbiota by promoting the growth of harmful bacteria while inhibiting the proliferation of beneficial bacteria, ultimately leading to damage in intestinal health and growth performance.

In this study, supplementing GUE in the diet contaminated with DON and ZEN significantly decreased the relative abundance of harmful bacterial genera (species) in cecum of broilers, including *Bacteroides* (*Bacteroides_fragilis* and *Bacteroides_dorei*), *Helicobacter* (*Helicobacter_pullorum*) and *Escherichia* (*Escherichia_coli*). Moreover, we found that GUE significantly increased the abundance of the genus *Lactobacillus* including *Lactobacillus_gallinarum* and *Lactobacillus_crispatus* in the cecum of broilers at 28 d of age, and *Alistipes* and *Bacteroides_vulgatus* at 84 d of age. Consistent with this, Li et al. (32) showed that dietary GUE decreased the abundance of *Bacteroides* (*B. fragilis* and *B. dore*), *Helicobacter* (*Helicobacter_pullorum*), and *Escherichia* (*Escherichia_coli*), while increasing that of *Lactobacillus* and *Lactobacillus gallinarum* in the cecum of broilers. Dietary supplementation with *Glycyrrhiza* polysaccharides suppressed the proliferation of *Bacteroides* (31). Lin and Lee (102) reported that *Lactobacillus* facilitated the development of an optimized microbiome by increasing the richness and quantity of *lactobacilli* and other native probiotic bacteria. *Lactobacillus* generates several proteolytic enzymes that detoxify mycotoxins, helping to alleviate cecal microbiota dysbiosis in broiler chickens (103). *Lactobacillus_crispatus* (104) and *Lactobacillus_gallinarum* (99) stimulated the growth of butyrate-producing microbiota and restored the balance of gut microbiota. Total flavonoids from *Glycyrrhiza uralensis* alleviated irinotecan-induced colitis and gut microbiota dysbiosis by enhancing populations of *Lactobacillus* and butyrate-producing *Roseburia* in mice (105). Therefore, it is reasonable to assume that GUE could regulate the balance of intestinal microecology, and effectively mitigate the damage caused by DON and ZEN to the broilers. However, the host and symbiotic gut microbiota are closely connected and interact with each other. Our experimental evidence was insufficient to clarify how GUE and the mycotoxins impacted broiler health through host-microbiota interactions.

## 5 Conclusion

In summary, the long-term consumption of low doses of DON and ZEN from naturally moldy corn reduced growth performance in broilers, particularly during the growing-finishing period, and compromised intestinal health by damaging intestinal morphology, barrier, and antioxidant functions, and inducing an inflammatory response in the jejunum. Furthermore, this chronic exposure to the mycotoxins disrupted the balance of intestinal microecology by increasing harmful microbiota, and decreasing beneficial microbiota in the cecum. The supplementation with GUE mitigated the negative effects of prolonged low-dose exposure to DON and ZEN on growth performance and intestinal health by improving the morphology, tight junction, antioxidant function, and inflammation in the jejunum, as well as the cecal microbiota balance in broilers.

## Data Availability

The datasets presented in this study can be found in online repositories. The names of the repository/repositories and accession number(s) can be found at: https://www.ncbi.nlm.nih.gov/, PRJNA1185367.

## Footnotes

^1^ www.biocloud.net

## References

[1] Dietrich B Neuenschwander S Bucher B Wenk C. Fusarium mycotoxin-contaminated wheat containing Deoxynivalenol alters the gene expression in the liver and the jejunum of broilers. Animal. (2012) 6:278–91. doi: 10.1017/s1751731111001601, PMID: 22436186

[2] Jones G Usher J Latham Rosie L Boyle Jeremy T Barbano A Loveman William G. Diverse mycotoxin threats to safe food and feed cereals. Essays Biochem. (2023) 67:797–809. doi: 10.1042/ebc20220221, PMID: 37313591 PMC10500202

[3] Da Costa RV Queiroz VAV Cota LV Da Silva DD Lanza FE De Almeida REM. Delaying harvest for naturally drying maize grain increases the risk of kernel rot and Fumonisin contamination. Trop Plant Pathol. (2018) 43:452–9. doi: 10.1007/s40858-018-0234-0

[4] Eskola M Kos G Elliott CT Hajslova J Mayar S Krska R. Worldwide contamination of food-crops with mycotoxins: validity of the widely cited “Fao Estimate” of 25. Crit Rev Food Sci Nutr. (2020) 60:2773–89. doi: 10.1080/10408398.2019.1658570, PMID: 31478403

[5] Ochieng PE Scippo ML Kemboi DC Croubels S Okoth S Kang'ethe EK. Mycotoxins in poultry feed and feed ingredients from sub-Saharan Africa and their impact on the production of broiler and layer chickens: a review. Toxins (Basel). (2021) 13:633. doi: 10.3390/toxins13090633, PMID: 34564637 PMC8473361

[6] Imade F Ankwasa EM Geng H Ullah S Ahmad T Wang G. Updates on food and feed mycotoxin contamination and safety in Africa with special reference to Nigeria. Mycology. (2021) 12:245–60. doi: 10.1080/21501203.2021.1941371, PMID: 34900380 PMC8654414

[7] Smith M-C Hymery N Troadec S Pawtowski A Coton E Madec S. Hepatotoxicity of Fusariotoxins, alone and in combination, towards the Heparg human hepatocyte cell line. Food Chem Toxicol. (2017) 109:439–51. doi: 10.1016/j.fct.2017.09.022, PMID: 28935499

[8] Ma R Zhang L Liu M Su YT Xie WM Zhang NY. Individual and combined occurrence of mycotoxins in feed ingredients and complete feeds in China. Toxins (Basel). (2018) 10:113. doi: 10.3390/toxins10030113, PMID: 29518909 PMC5869401

[9] Che Z Liu Y Wang H Zhu H Hou Y Ding B. The protective effects of different mycotoxin adsorbents against blood and liver pathological changes induced by mold-contaminated feed in broilers. Asian-Australas J Anim Sci. (2011) 24:250–7. doi: 10.5713/ajas.2011.10022

[10] Awad W Ghareeb K Bohm J Zentek J. The toxicological impacts of the Fusarium mycotoxin, Deoxynivalenol, in poultry flocks with special reference to Immunotoxicity. Toxins (Basel). (2013) 5:912–25. doi: 10.3390/toxins5050912, PMID: 23628787 PMC3709269

[11] Schoevers EJ Fink-Gremmels J Colenbrander B Roelen BA. Porcine oocytes are Most vulnerable to the mycotoxin Deoxynivalenol during formation of the meiotic spindle. Theriogenology. (2010) 74:968–78. doi: 10.1016/j.theriogenology.2010.04.026, PMID: 20570324

[12] Rajashekara G Antonissen G Van Immerseel F Pasmans F Ducatelle R Haesebrouck F. The mycotoxin Deoxynivalenol predisposes for the development of *Clostridium Perfringens*-induced necrotic enteritis in broiler chickens. PLoS One. (2014) 9:e108775. doi: 10.1371/journal.pone.0108775, PMID: 25268498 PMC4182565

[13] Feng W Liu Y Ding Y Mao G Zhao T Chen K. Typical neurobehavioral methods and transcriptome analysis reveal the neurotoxicity and mechanisms of Di(2-Ethylhexyl) phthalate on pubertal male Icr mice with type 2 diabetes mellitus. Arch Toxicol. (2020) 94:1279–302. doi: 10.1007/s00204-020-02683-9, PMID: 32303808

[14] Santos L Marín S Sanchis V Ramos AJ. In vitro effect of some fungicides used in cultivation of Capsicum Spp. on growth and Ochratoxin a production Byaspergillus species. World Mycotoxin J. (2013) 6:159–66. doi: 10.3920/wmj2012.1480

[15] Przybylska-Gornowicz B Lewczuk B Prusik M Hanuszewska M Petrusewicz-Kosińska M Gajęcka M. The effects of Deoxynivalenol and Zearalenone on the pig large intestine. A light and electron microscopy study. Toxins. (2018) 10:148. doi: 10.3390/toxins10040148, PMID: 29617295 PMC5923314

[16] Vanhoutte I Audenaert K De Gelder L. Biodegradation of mycotoxins: Tales from known and unexplored worlds. Front Microbiol. (2016) 7:7. doi: 10.3389/fmicb.2016.00561, PMID: 27199907 PMC4843849

[17] Wang W Zhu J Cao Q Zhang C Dong Z Feng D. Dietary catalase supplementation alleviates Deoxynivalenol-induced oxidative stress and gut microbiota Dysbiosis in broiler chickens. Toxins. (2022) 14:830. doi: 10.3390/toxins14120830, PMID: 36548727 PMC9784562

[18] Raei H Najafi R Torshizi MAK Froushani SMA Joogh FAG. The effect of *Silybum Marianum* seed, thymus vulgaris, and *Rosmarinus Officinalis* powders in alleviating the risks of aflatoxin B1 in young broiler chicks. Ann Anim Sci. (2022) 22:173–87. doi: 10.2478/aoas-2021-0027

[19] Xu J Li S Jiang L Gao X Liu W Zhu X. Baicalin protects against Zearalenone-induced chicks liver and kidney injury by inhibiting expression of oxidative stress, inflammatory cytokines and caspase signaling pathway. Int Immunopharmacol. (2021) 100:100. doi: 10.1016/j.intimp.2021.108097, PMID: 34521024

[20] Yuanpei Lei JZ Zheng WG Zhao L Cheng JI. A survey report on the mycotoxin contamination of compound feed and its raw materials of China in 2019-2020. Siliao Gongye. (2022) 43:59–64. doi: 10.13302/j.cnki.fi.2022.20.010

[21] Ghareeb K Awad WA Soodoi C Sasgary S Strasser A Böhm J. Effects of feed contaminant Deoxynivalenol on plasma cytokines and Mrna expression of immune genes in the intestine of broiler chickens. PLoS One. (2013) 8:e71492. doi: 10.1371/journal.pone.0071492, PMID: 23977054 PMC3748120

[22] Pinton P Oswald IP. Effect of Deoxynivalenol and other type B Trichothecenes on the intestine: a review. Toxins (Basel). (2014) 6:1615–43. doi: 10.3390/toxins6051615, PMID: 24859243 PMC4052256

[23] Yang R Yuan BC Ma YS Zhou S Liu Y. The anti-inflammatory activity of licorice, a widely used Chinese herb. Pharm Biol. (2017) 55:5–18. doi: 10.1080/13880209.2016.1225775, PMID: 27650551 PMC7012004

[24] Reda FM El-Saadony MT El-Rayes TK Farahat M Attia G Alagawany M. Dietary effect of licorice (*Glycyrrhiza Glabra*) on quail performance, carcass, blood metabolites and intestinal microbiota. Poult Sci. (2021) 100:101266. doi: 10.1016/j.psj.2021.101266, PMID: 34225203 PMC8264150

[25] Ayeka PA Bian Y Githaiga PM Zhao Y. The immunomodulatory activities of licorice polysaccharides (Glycyrrhiza Uralensis Fisch.) in Ct 26 tumor-bearing mice. BMC Complement Altern Med. (2017) 17:536. doi: 10.1186/s12906-017-2030-7, PMID: 29246138 PMC5732493

[26] Wittschier N Faller G Hensel A. Aqueous extracts and polysaccharides from Liquorice roots (*Glycyrrhiza Glabra* L.) inhibit adhesion of *Helicobacter Pylori* to human gastric mucosa. J Ethnopharmacol. (2009) 125:218–23. doi: 10.1016/j.jep.2009.07.009, PMID: 19607905

[27] Rozi P Abuduwaili A Ma SJ Bao XW Xu HZX Zhu JF. Isolations, characterizations and bioactivities of polysaccharides from the seeds of three species Glycyrrhiza. Int J Biol Macromol. (2020) 145:364–71. doi: 10.1016/j.ijbiomac.2019.12.107, PMID: 31857172

[28] Ibrahim D Sewid AH Arisha AH Abd El-Fattah AH Abdelaziz AM Al-Jabr OA. Influence of *Glycyrrhiza Glabra* extract on growth, gene expression of gut integrity, and *Campylobacter Jejuni* colonization in broiler chickens. Front Vet Sci. (2020) 7:612063. doi: 10.3389/fvets.2020.612063, PMID: 33415133 PMC7782238

[29] Wang X Zhao P Zhang C Li C Ma Y Huang S. Effects of supplemental Glycyrrhiza polysaccharide on growth performance and intestinal health in weaned piglets. Anim Biotechnol. (2024) 35:2362640. doi: 10.1080/10495398.2024.2362640, PMID: 38860902 PMC12674271

[30] Bachinger D Mayer E Kaschubek T Schieder C Konig J Teichmann K. Influence of Phytogenics on recovery of the barrier function of intestinal porcine epithelial cells after a calcium switch. J Anim Physiol Anim Nutr (Berl). (2019) 103:210–20. doi: 10.1111/jpn.12997, PMID: 30353576 PMC7379982

[31] Qiao Y Liu C Guo Y Zhang W Guo W Oleksandr K. Polysaccharides derived from Astragalus Membranaceus and Glycyrrhiza Uralensis improve growth performance of broilers by enhancing intestinal health and modulating gut microbiota. Poult Sci. (2022) 101:101905. doi: 10.1016/j.psj.2022.101905, PMID: 35576745 PMC9117935

[32] Li X Li J Yuan H Chen Y Li S Jiang S. Effect of supplementation with Glycyrrhiza Uralensis extract and *Lactobacillus Acidophilus* on growth performance and intestinal health in broiler chickens. Front Vet Sci. (2024) 11:1436807. doi: 10.3389/fvets.2024.1436807, PMID: 39091388 PMC11291472

[33] Grenier B Applegate TJ. Modulation of intestinal functions following mycotoxin ingestion: Meta-analysis of published experiments in animals. Toxins (Basel). (2013) 5:396–430. doi: 10.3390/toxins5020396, PMID: 23430606 PMC3640542

[34] Jung JC Lee YH Kim SH Kim KJ Kim KM Oh S. Hepatoprotective effect of licorice, the root of Glycyrrhiza Uralensis Fischer, in alcohol-induced fatty liver disease. BMC Complement Altern Med. (2016) 16:19. doi: 10.1186/s12906-016-0997-0, PMID: 26801973 PMC4722619

[35] Bryden WL. Mycotoxin contamination of the feed supply chain: implications for animal productivity and feed security. Anim Feed Sci Technol. (2012) 173:134–58. doi: 10.1016/j.anifeedsci.2011.12.014

[36] Zou Y Yang ZB Yang WR Jiang SZ Zhang GG Chi F. Effect of purified Zearalenone on nutrient digestibility in broilers fed 2 levels of Fumonisin from naturally contaminated corn (*Zea Mays*). J Appl Poult Res. (2012) 21:251–8. doi: 10.3382/japr.2011-00359

[37] Chen Y Cheng Y Wen C Wang W Kang Y Wang A. The protective effects of modified Palygorskite on the broilers fed a purified Zearalenone-contaminated diet. Poult Sci. (2019) 98:3802–10. doi: 10.3382/ps/pez085, PMID: 30839081

[38] Awad WA Hess M Twaruzek M Grajewski J Kosicki R Bohm J. The impact of the Fusarium mycotoxin Deoxynivalenol on the health and performance of broiler chickens. Int J Mol Sci. (2011) 12:7996–8012. doi: 10.3390/ijms12117996, PMID: 22174646 PMC3233452

[39] Peillod C Laborde M Travel A Mika A Bailly JD Cleva D. Toxic effects of Fumonisins, Deoxynivalenol and Zearalenone alone and in combination in ducks fed the maximum Eutolerated level. Toxins. (2021) 13:152. doi: 10.3390/toxins13020152 33669302 PMC7920068

[40] Tardieu D Travel A Le Bourhis C Metayer JP Mika A Cleva D. Fumonisins and Zearalenone fed at low levels can persist several days in the liver of turkeys and broiler chickens after exposure to the contaminated diet was stopped. Food Chem Toxicol. (2021) 148:111968. doi: 10.1016/j.fct.2021.111968, PMID: 33422601

[41] Abo-Samaha MI Alghamdi YS El-Shobokshy SA Albogami S El-Maksoud EMA Farrag F. Licorice extract supplementation affects antioxidant activity, growth-related genes, lipid metabolism, and immune markers in broiler chickens. Life. (2022) 12:914. doi: 10.3390/life12060914, PMID: 35743945 PMC9225592

[42] Salary J Kalantar M Ala M Ranjbar K Matin HH. Drinking water supplementation of licorice and *Aloe Vera* extracts in broiler chickens. Sci J Anim Sci. (2014) 3:41–8. doi: 10.14196/sjas.v3i2.1144

[43] Beski SSM Shekhu NA Sadeq SAM AL-Khdri AM. Effects of the addition of aqueous Liquorice (*Glycyrrhiza Glabra*) extract to drinking water in the production performance, carcass cuts and intestinal Histomorphology of broiler chickens. Iraqi J Agric Sci. (2019) 50:842–9. doi: 10.36103/ijas.v50i3.701

[44] Riahi I Marquis V Perez-Vendrell AM Brufau J Esteve-Garcia E Ramos AJ. Effects of Deoxynivalenol-contaminated diets on metabolic and immunological parameters in broiler chickens. Animals (Basel). (2021) 11:147. doi: 10.3390/ani11010147, PMID: 33440734 PMC7826962

[45] Ghareeb K Awad WA Bohm J Zebeli Q. Impacts of the feed contaminant Deoxynivalenol on the intestine of Monogastric animals: poultry and swine. J Appl Toxicol. (2015) 35:327–37. doi: 10.1002/jat.3083, PMID: 25352520

[46] Awad WA Böhm J Razzazi-Fazeli E Ghareeb K Zentek J. Effect of addition of a probiotic microorganism to broiler diets contaminated with Deoxynivalenol on performance and histological alterations of intestinal villi of broiler chickens. Poult Sci. (2006) 85:974–9. doi: 10.1093/ps/85.6.974, PMID: 16776464

[47] Zhang C Chen KK Zhao XH Wang C Geng ZY. Effect of L-Theanine on the growth performance, immune function, and jejunum morphology and antioxidant status of ducks. Animal. (2019) 13:1145–53. doi: 10.1017/S1751731118002884, PMID: 30376911

[48] Zhao W Chen Y Tian Y Wang Y Du J Ye X. Dietary supplementation with Dendrobium Officinale leaves improves growth, antioxidant status, immune function, and gut health in broilers. Front Microbiol. (2023) 14:1255894. doi: 10.3389/fmicb.2023.1255894, PMID: 37789853 PMC10544969

[49] Wu Y Zhang H Zhang R Cao G Li Q Zhang B. Serum metabolome and gut microbiome alterations in broiler chickens supplemented with Lauric acid. Poult Sci. (2021) 100:101315. doi: 10.1016/j.psj.2021.101315, PMID: 34280650 PMC8318919

[50] Liu K Jia M Wong EA. Delayed access to feed affects broiler small intestinal morphology and goblet cell ontogeny. Poult Sci. (2020) 99:5275–85. doi: 10.1016/j.psj.2020.07.040, PMID: 33142443 PMC7647802

[51] Wan S Sun N Li H Khan A Zheng X Sun Y. Deoxynivalenol damages the intestinal barrier and biota of the broiler chickens. BMC Vet Res. (2022) 18:311. doi: 10.1186/s12917-022-03392-4, PMID: 35965338 PMC9377127

[52] Gyawali I Zeng Y Zhou J Li J Wu T Shu G. Effect of novel Lactobacillus Paracaesi microcapsule on growth performance, gut health and microbiome Community of Broiler Chickens. Poult Sci. (2022) 101:101912. doi: 10.1016/j.psj.2022.101912, PMID: 35689995 PMC9190013

[53] Mohammed E Kamel M El Iraqi K Tawfik AM Khattab MS Elsabagh M. Zingiber Officinale and *Glycyrrhiza Glabra*, individually or in combination, reduce heavy metal accumulation and improve growth performance and immune status in Nile Tilapia, *Oreochromis Niloticus*. Aquac Res. (2020) 51:1933–41. doi: 10.1111/are.14544

[54] Jia S Ren C Yang P Qi D. Effects of intestinal microorganisms on metabolism and toxicity mitigation of Zearalenone in broilers. Animals. (2022) 12:1962. doi: 10.3390/ani12151962, PMID: 35953951 PMC9367588

[55] Wei X Li N Wu X Cao G Qiao H Wang J. The preventive effect of Glycyrrhiza polysaccharide on lipopolysaccharide-induced acute colitis in mice by modulating gut microbial communities. Int J Biol Macromol. (2023) 239:124199. doi: 10.1016/j.ijbiomac.2023.124199, PMID: 36972824

[56] Zhang C Li C Zhao P Shao Q Ma Y Bai D. Effects of dietary Glycyrrhiza polysaccharide supplementation on growth performance, intestinal antioxidants, immunity and microbiota in weaned piglets. Anim Biotechnol. (2023) 34:2273–84. doi: 10.1080/10495398.2022.2086878, PMID: 35714985

[57] Vancamelbeke M Vermeire S. The intestinal barrier: a fundamental role in health and disease. Expert Rev Gastroenterol Hepatol. (2017) 11:821–34. doi: 10.1080/17474124.2017.1343143, PMID: 28650209 PMC6104804

[58] Heinemann U Schuetz A. Structural features of tight-junction proteins. Int J Mol Sci. (2019) 20:6020. doi: 10.3390/ijms20236020, PMID: 31795346 PMC6928914

[59] Suzuki T. Regulation of intestinal epithelial permeability by tight junctions. Cell Mol Life Sci. (2013) 70:631–59. doi: 10.1007/s00018-012-1070-x, PMID: 22782113 PMC11113843

[60] Pinton P Nougayrede JP Del Rio JC Moreno C Marin DE Ferrier L. The food contaminant Deoxynivalenol, decreases intestinal barrier permeability and reduces Claudin expression. Toxicol Appl Pharmacol. (2009) 237:41–8. doi: 10.1016/j.taap.2009.03.003, PMID: 19289138

[61] Lai Y Sun M He Y Lei J Han Y Wu Y. Mycotoxins binder supplementation alleviates aflatoxin B1 toxic effects on the immune response and intestinal barrier function in broilers. Poult Sci. (2022) 101:101683. doi: 10.1016/j.psj.2021.101683, PMID: 35121530 PMC8883060

[62] Lee SH Kwon JE Cho ML. Immunological pathogenesis of inflammatory bowel disease. Intest Res. (2018) 16:26–42. doi: 10.5217/ir.2018.16.1.26, PMID: 29422795 PMC5797268

[63] Cano PM Seeboth J Meurens F Cognie J Abrami R Oswald IP. Deoxynivalenol as a new factor in the persistence of intestinal inflammatory diseases: an emerging hypothesis through possible modulation of Th17-mediated response. PLoS One. (2013) 8:e53647. doi: 10.1371/journal.pone.0053647, PMID: 23326479 PMC3542340

[64] Gocher AM Workman CJ Vignali DAA. Interferon-Γ: teammate or opponent in the tumour microenvironment? Nat Rev Immunol. (2022) 22:158–72. doi: 10.1038/s41577-021-00566-3, PMID: 34155388 PMC8688586

[65] Utech M Ivanov AI Samarin SN Bruewer M Turner JR Mrsny RJ. Mechanism of Ifn-gamma-induced endocytosis of tight junction proteins: myosin ii-dependent Vacuolarization of the apical plasma membrane. Mol Biol Cell. (2005) 16:5040–52. doi: 10.1091/mbc.e05-03-0193, PMID: 16055505 PMC1237102

[66] Li H Wang X Pan H Xiao C Wang C Guo S. The mechanisms and functions of Il-1β in intervertebral disc degeneration. Exp Gerontol. (2023) 177:112181. doi: 10.1016/j.exger.2023.112181, PMID: 37088216

[67] Jia R Liu W Zhao L Cao L Shen Z. Low doses of individual and combined Deoxynivalenol and Zearalenone in naturally moldy diets impair intestinal functions via inducing inflammation and disrupting epithelial barrier in the intestine of piglets. Toxicol Lett. (2020) 333:159–69. doi: 10.1016/j.toxlet.2020.07.032, PMID: 32783910

[68] Grenier B Dohnal I Shanmugasundaram R Eicher SD Selvaraj RK Schatzmayr G. Susceptibility of broiler chickens to coccidiosis when fed subclinical doses of Deoxynivalenol and Fumonisins-special emphasis on the immunological response and the mycotoxin interaction. Toxins. (2016) 8:231. doi: 10.3390/toxins8080231, PMID: 27472362 PMC4999847

[69] Sharifi-Rad J Quispe C Herrera-Bravo J Belén LH Kaur R Kregiel D. Glycyrrhiza genus: enlightening phytochemical components for pharmacological and health-promoting abilities. Oxidative Med Cell Longev. (2021) 2021:2021(1-20). doi: 10.1155/2021/7571132, PMID: 34349875 PMC8328722

[70] Murugan SK Bethapudi B Raghunandhakumar S Purusothaman D Nithyanantham M Mundkinajeddu D. A flavonoid rich standardized extract of *Glycyrrhiza Glabra* protects intestinal epithelial barrier function and regulates the tight-junction proteins expression. BMC Complement Med Ther. (2022) 22:38. doi: 10.1186/s12906-021-03500-1, PMID: 35130890 PMC8822647

[71] Mohd Shaufi MA Sieo CC Chong CW Gan HM Ho YW. Deciphering chicken gut microbial dynamics based on high-throughput 16s Rrna metagenomics analyses. Gut Pathog. (2015) 7:4. doi: 10.1186/s13099-015-0051-7, PMID: 25806087 PMC4372169

[72] Han GG Kim EB Lee J Lee JY Jin G Park J. Relationship between the microbiota in different sections of the gastrointestinal tract, and the body weight of broiler chickens. Springerplus. (2016) 5:911. doi: 10.1186/s40064-016-2604-8, PMID: 27386355 PMC4927549

[73] Clavijo V Florez MJV. The gastrointestinal microbiome and its association with the control of pathogens in broiler chicken production: a review. Poult Sci. (2018) 97:1006–21. doi: 10.3382/ps/pex359, PMID: 29253263 PMC5850219

[74] Lucke A Böhm J Zebeli Q Metzler-Zebeli BU. Dietary Deoxynivalenol contamination and Oral lipopolysaccharide challenge alters the Cecal microbiota of broiler chickens. Front Microbiol. (2018) 9:9. doi: 10.3389/fmicb.2018.00804, PMID: 29922239 PMC5996912

[75] Wu Y Wu C Che Y Zhang T Dai C Nguyen AD. Effects of Glycyrrhiza polysaccharides on Chickens' intestinal health and homeostasis. Front Vet Sci. (2022) 9:891429. doi: 10.3389/fvets.2022.891429, PMID: 35647094 PMC9134109

[76] Pastor-Fernandez I Pegg E Macdonald SE Tomley FM Blake DP Marugan-Hernandez V. Laboratory growth and genetic manipulation of Eimeria Tenella. Curr Protoc Microbiol. (2019) 53:e81. doi: 10.1002/cpmc.81, PMID: 30811108

[77] Han GG Lee JY Jin GD Park J Choi YH Chae BJ. Evaluating the association between body weight and the intestinal microbiota of weaned piglets via 16s Rrna sequencing. Appl Microbiol Biotechnol. (2017) 101:5903–11. doi: 10.1007/s00253-017-8304-7, PMID: 28523395

[78] Salaheen S Kim SW Haley BJ Van Kessel JAS Biswas D. Alternative growth promoters modulate broiler gut microbiome and enhance body weight gain. Front Microbiol. (2017) 8:2088. doi: 10.3389/fmicb.2017.02088, PMID: 29123512 PMC5662582

[79] Juan Changa TW Wanga P Yina Q Liua C Zhub Q Luc F. Compound probiotics alleviating aflatoxin B1 and Zearalenone toxic effects on broiler production performance and gut microbiota. Ecotoxicol Environ Saf. (2020) 194:110420. doi: 10.1016/j.ecoenv.2020.110420, PMID: 32151861

[80] Kang MH Jang GY Ji YJ Lee JH Choi SJ Hyun TK. Antioxidant and anti-melanogenic activities of heat-treated licorice (Wongam, *Glycyrrhiza Glabra* X *G. uralensis*) extract. Curr Issues Mol Biol. (2021) 43:1171–87. doi: 10.3390/cimb43020083, PMID: 34563052 PMC8928971

[81] Qiao Y Guo Y Zhang W Guo W Oleksandr K Bozhko N. Effects of compound polysaccharides derived from Astragalus and Glycyrrhiza on growth performance, meat quality and antioxidant function of broilers based on serum metabolomics and Cecal microbiota. Antioxidants (Basel). (2022) 11:1872. doi: 10.3390/antiox11101872, PMID: 36290595 PMC9598874

[82] Ma X Wang Q Li H Xu C Cui N Zhao X. 16s Rrna genes Illumina sequencing revealed differential Cecal microbiome in specific pathogen free chickens infected with different subgroup of avian Leukosis viruses. Vet Microbiol. (2017) 207:195–204. doi: 10.1016/j.vetmic.2017.05.016, PMID: 28757024

[83] Oakley BB Kogut MH. Spatial and temporal changes in the broiler chicken Cecal and fecal microbiomes and correlations of bacterial taxa with cytokine gene expression. Front Vet Sci. (2016) 3:3. doi: 10.3389/fvets.2016.00011, PMID: 26925404 PMC4759570

[84] Waite DW Vanwonterghem I Rinke C Parks DH Zhang Y Takai K. Comparative genomic analysis of the class Epsilonproteobacteria and proposed reclassification to Epsilonbacteraeota (Phyl Nov.). Front Microbiol. (2017) 8:682. doi: 10.3389/fmicb.2017.00682, PMID: 28484436 PMC5401914

[85] Javed S Gul F Javed K Bokhari H. *Helicobacter Pullorum*: an emerging zoonotic pathogen. Front Microbiol. (2017) 8:604. doi: 10.3389/fmicb.2017.00604, PMID: 28443081 PMC5385324

[86] Shin NR Whon TW Bae JW. Proteobacteria: microbial signature of Dysbiosis in gut microbiota. Trends Biotechnol. (2015) 33:496–503. doi: 10.1016/j.tibtech.2015.06.011 26210164

[87] Borrelli L Varriale L Coretti L Pace A Russo TP Santaniello A. Research note: Cecal microbiota harbored by free-range chickens may influence the reduction of *Helicobacter Pullorum* relative abundance. Poult Sci. (2023) 102:102222. doi: 10.1016/j.psj.2022.102222, PMID: 36502562 PMC9763842

[88] Abdel-Shafi S Abd El-Hack ME Amen S Helmi A Swelum AA Tellez-Isaias G. The efficacy of some probiotics and prebiotics on the prevalence of E. Coli and the immune response of chickens. Poult Sci. (2023) 102:103219. doi: 10.1016/j.psj.2023.103219, PMID: 37993387 PMC10755822

[89] Zafar H Saier MH Jr. Gut Bacteroides species in health and disease. Gut Microbes. (2021) 13:1–20. doi: 10.1080/19490976.2020.1848158, PMID: 33535896 PMC7872030

[90] Rubio LA Peinado MJ Ruiz R Suarez-Pereira E Ortiz Mellet C Garcia Fernandez JM. Correlations between changes in intestinal microbiota composition and performance parameters in broiler chickens. J Anim Physiol Anim Nutr (Berl). (2015) 99:418–23. doi: 10.1111/jpn.12256, PMID: 25266875

[91] Zakharzhevskaya NB Vanyushkina AA Altukhov IA Shavarda AL Butenko IO Rakitina DV. Outer membrane vesicles secreted by pathogenic and nonpathogenic *Bacteroides Fragilis* represent different metabolic activities. Sci Rep. (2017) 7:5008. doi: 10.1038/s41598-017-05264-6, PMID: 28694488 PMC5503946

[92] Gong L Wang B Zhou Y Tang L Zeng Z Zhang H. Protective effects of *Lactobacillus Plantarum* 16 and *Paenibacillus Polymyxa* 10 against *Clostridium Perfringens* infection in broilers. Front Immunol. (2020) 11:628374. doi: 10.3389/fimmu.2020.628374, PMID: 33679724 PMC7930238

[93] Gruber-Dorninger C Jenkins T Schatzmayr G. Global mycotoxin occurrence in feed: a ten-year survey. Toxins. (2019) 11:375. doi: 10.3390/toxins11070375, PMID: 31252650 PMC6669473

[94] Wu Y Lei Z Wang Y Yin D Aggrey SE Guo Y. Metabolome and microbiota analysis reveals the conducive effect of *Pediococcus Acidilactici* Bcc-1 and Xylan oligosaccharides on broiler chickens. Front Microbiol. (2021) 12:12. doi: 10.3389/fmicb.2021.683905, PMID: 34122394 PMC8192963

[95] Zhang M Mo R Wang H Liu T Zhang G Wu Y. Grape seed Proanthocyanidin improves intestinal inflammation in canine through regulating gut microbiota and bile acid compositions. FASEB J. (2023) 37:e23285. doi: 10.1096/fj.202300819RR, PMID: 37933950

[96] Yang F Li J Wei L Qin S Shi Q Lu S. The characteristics of intestinal microbiota in patients with type 2 diabetes and the correlation with the percentage of T-helper cells. Front Microbiol. (2024) 15:1443743. doi: 10.3389/fmicb.2024.1443743, PMID: 39397795 PMC11466775

[97] Wu X Xu J Li J Deng M Shen Z Nie K. *Bacteroides Vulgatus* alleviates dextran sodium sulfate-induced colitis and depression-like behaviour by facilitating gut-brain Axis balance. Front Microbiol. (2023) 14:1287271. doi: 10.3389/fmicb.2023.1287271, PMID: 38033588 PMC10687441

[98] Tannock GW. A special fondness for lactobacilli. Appl Environ Microbiol. (2004) 70:3189–94. doi: 10.1128/aem.70.6.3189-3194.2004, PMID: 15184111 PMC427720

[99] Sugimura N Li Q Chu ESH Lau HCH Fong W Liu W. *Lactobacillus Gallinarum* modulates the gut microbiota and produces anti-Cancer metabolites to protect against colorectal Tumourigenesis. Gut. (2021) 71:2011–21. doi: 10.1136/gutjnl-2020-323951, PMID: 34937766 PMC9484392

[100] Khan M. Effect of *Lactobacillus Gallinarum* Pl 53 supplementation on xylose absorption and intestinal morphology in broilers challenged with *Campylobacter Jejuni*. Pak Vet J. (2019) 40:168–8. doi: 10.29261/pakvetj/2020.011

[101] Neveling DP van Emmenes L Ahire JJ Pieterse E Smith C Dicks LMT. Effect of a multi-species probiotic on the colonisation of Salmonella in broilers. Probiotics Antimicrob Proteins. (2020) 12:896–905. doi: 10.1007/s12602-019-09593-y, PMID: 31784950

[102] Lin WC Lee TT. Effects of Laetiporus Sulphureus-fermented wheat bran on growth performance, intestinal microbiota and Digesta characteristics in broiler chickens. Animals. (2020) 10:1457. doi: 10.3390/ani10091457, PMID: 32825244 PMC7552699

[103] Shanmugasundaram R Lourenco J Hakeem WA Dycus MM Applegate TJ. Subclinical doses of dietary Fumonisins and Deoxynivalenol cause Cecal microbiota Dysbiosis in broiler chickens challenged with *Clostridium Perfringens*. Front Microbiol. (2023) 14:14. doi: 10.3389/fmicb.2023.1106604, PMID: 37082176 PMC10111830

[104] De Maesschalck C Eeckhaut V Maertens L De Lange L Marchal L Nezer C. Effects of Xylo-oligosaccharides on broiler chicken performance and microbiota. Appl Environ Microbiol. (2015) 81:5880–8. doi: 10.1128/AEM.01616-15, PMID: 26092452 PMC4551243

[105] Yue SJ Qin YF Kang A Tao HJ Zhou GS Chen YY. Total flavonoids of Glycyrrhiza Uralensis alleviates Irinotecan-induced colitis via modification of gut microbiota and fecal metabolism. Front Immunol. (2021) 12:628358. doi: 10.3389/fimmu.2021.628358, PMID: 34025639 PMC8138048

